# Supplementary material for: Stimulatory Effects of Extracellular Vesicles Derived from Leuconostoc holzapfelii That Exists in Human Scalp on Hair Growth in Human Follicle Dermal Papilla Cells
Source: Curr Issues Mol Biol. 2022 Feb 10;44(2):845–66. doi: 10.3390/cimb44020058 (PMC8929027; doi:10.3390/cimb44020058)
Supplement: Supplementary file 1 [file cimb-44-00058-s001.zip › cimb-1530104-supplementary Eng.pdf]

Clinical Test Report

GFC Life Science Co.,Ltd.

Double-blind, placebo-controlled, single-site, randomized,

human application test to evaluate safety and

effectiveness of alopecia prevention of

"Leuco-Exo (sample 1) & Hair solvent (sample 2)"

Date: 07/30/2021

Korea Dermatology Research Institute

KDRI Korea Dermatology Research Institute

TABLE OF CONTENT

1. Summary of Test Result ······3
2. Quality Assurance Certificate ······4
3. Background of Evaluation Test ······8
4. Purpose of Evaluation Test ······8
5. Test Sample Identification ······9
6. Selection of Subjects ······10
7. Test Method ······12
8. Test Results ······15
9. Discussion and Conclusions ······20
10. References ······21

Attachment 1. Information of Test institution ······22
Attachment 2. Information of Institutional Review Board (IRB) ······25
Attachment 3. Information Provided to the Subjects ······26
Attachment 4. Compensation for Research Related Injuries ······29
Attachment 5. Appendix Image Data ······31

Korea Dermatology Research Institute

The contents of this report are confidential and cannot be used for other purposes unless approved.
All or any part of the contents in this report cannot be copied or published without consent of KDRI.

Summary of Test Result

<table>
<tr><td>TITLE</td><td colspan="4">Double-blind, placebo-controlled, single-site, randomized, human application test to evaluate safety and effectiveness of alopecia prevention of “Leuco-Exo (sample 1) & Hair solvent (sample 2)”</td></tr>
<tr><td>CLIENT</td><td colspan="4">GFC Life Science Co.,Ltd. (Person in Charge : Yoon Yeo Cho)
1708, Apexcity, 823, Dongtansunhwan-daero, Hwaseong-si, Gyeonggi-do, 18471, Rep.of Korea
(Tel. +82-31-5183-5541 / Fax. +82-31-5183-5519)</td></tr>
<tr><td>INSTITUTE</td><td colspan="4">Korea Dermatology Research Institute
5~8F, 98, Yatap-ro, Bundang-gu, Seongnam-si, Gyeonggi-do, Republic of Korea
(Tel. +82-31-704-0099 / Fax. +82-31-701-0189)</td></tr>
<tr><td>PERIOD</td><td colspan="2">Mar. 24. 2021 ~ Jul. 30. 2020</td><td>Test Period</td><td>Mar. 29. 2019 ~ Jul. 06. 2020</td></tr>
<tr><td rowspan="3">TEST METHOD</td><td>Number of Sample</td><td>2 EA (Test Sample, Placebo Sample)</td><td>Number of Subjects</td><td>31 (Final subject)</td></tr>
<tr><td>Treatment</td><td colspan="3">Self-application by subjects</td></tr>
<tr><td>Details on Test Method</td><td colspan="3">1. Subject Selection
: 31 subjects who met the selection criteria and were not included in exemption criteria were selected
2. Test Area
: Scalp
3. Application Method
: Self-application by subjects on the scalp two a week
4. Evaluation Method
a. Hair density and diameter change assessment by phototrichogram
b. Visual evaluation by researchers
c. Self-assessment by subjects
d. Cutaneous irritation evaluation by dermatologist</td></tr>
</table>

KDRI Korea Dermatology Research Institute

The contents of this report are confidential and cannot be used for other purposes unless approved.
All or any part of the contents in this report cannot be copied or published without consent of KDRI.

**TEST RESULT**	As a result of testing 31 research subjects suffering from hair loss during the 12-week test period, the areas where GFC Life Science Co.,Ltd. "Leuco-Exo (sample 1) & Hair solvent (sample 2)" were applied showed a statistically significant increase (p<0.05) in the number of hairs (N/cm^2), the main efficacy index, from Week 4 compared to before the test. On the other hand, the areas where the placebo sample had been applied showed no statistically significant changes (p<0.05) in the number of hairs. In addition, the comparison and interaction analysis of the weekly changes in the number of hairs between the groups revealed statistically significant differences (p<0.05). There were also statistically significant differences in changes in the number of hairs after Weeks 4, 8 and 12. Therefore, **"Leuco-Exo (sample 1) & Hair solvent (sample 2)" is judged to have alopecia prevention efficacy**. In addition, no specific adverse reactions were observed in any of the study subjects during the test. (There may be temporary changes and individual differences.)

KDRI Korea Dermatology Research Institute

The contents of this report are confidential and cannot be used for other purposes unless approved.
All or any part of the contents in this report cannot be copied or published without consent of KDRI.

2. Quality Assurance Certificate

- **TITLE** Double-blind, placebo-controlled, single-site, randomized, human application test to evaluate safety and effectiveness of alopecia prevention of "Leuco-Exo (sample 1) & Hair solvent (sample 2)"
- **TEST Number** KDRI-2020-880
- **IRB Approval Number** KDRI-IRB-20880

This study was conducted in good faith in accordance with the test plan based on the Standard Operating Procedure (SOP) of the Korea Dermatology Research Institute under the supervision of the principal investigator. All test results obtained during the test period are recorded on this report without any omission, the entire process of this study has been examined by the reliability assurance officer and given final approval from the principal investigator.

Inspection	Inspection Details	Date of Inspection	Reporting Date to Research Director
Facilities	1. Inspection of test equipment and facilities	2021.03.24	2021.03.24
Procedure	2. Inspection of the Test Plan	2021.03.24	2021.03.24
Test	3. Inspection of the management of research subjects	2021.07.30	2021.07.30
	4. Inspection during the main test	2021.07.30	2021.07.30
	5. Inspection during the result analysis step	2021.07.30	2021.07.30
	6. Inspection of the final report	2021.07.30	2021.07.30
Inspection of documentation		2021.07.30	2021.07.30

* Refer to the next page for inspection details

Revisions to this report were processed as follows at the request of the sponsor, and the sponsor is responsible for the changes.

No. of Amendment	Amendment Approval Date	Amendment Contents
0	None	None

The contents of this report are confidential and cannot be used for other purposes unless approved.
All or any part of the contents in this report cannot be copied or published without consent of KDRI.

Confirmation of Reliability Assurance

Inspection	Details	
Facilities	**1. Test equipment and facilities**	**Inspection result**
	1-1. Are the test facilities adequately sized, structured, and arranged to meet research needs?	■ Yes ☐ No
	1-2. Are there facilities in place for the receipt and storage of test and control samples and excipients and test samples each to prevent contamination or confusion?	■ Yes ☐ No
	1-3. Are the rooms or areas storing test samples adequate to maintain/preserve identity, concentration, purity, and stability?	■ Yes ☐ No
	1-4. Are there facilities to safely store hazardous substances?	■ Yes ☐ No
	1-5. Are there storage facilities for the storage and retrieval of related documents, such as test plans, basic test data, and final reports?	■ Yes ☐ No
	1-6. Are SOPs in place for the use and operation of test equipment and facilities?	■ Yes ☐ No
Procedures	**2. Review of Test Plan**	**Inspection result**
	2-1. Does it include the test title, purpose, and number?	■ Yes ☐ No
	2-2. Does it include the sponsor and test institution?	■ Yes ☐ No
	2-3. Does it include the investigator's CV?	■ Yes ☐ No
	2-4. Does it include sample code numbers and properties and information on the components?	■ Yes ☐ No
	2-5. Are the test period and method clear and specific?	■ Yes ☐ No
	2-6. Are the research periods and analysis methods clear and specific?	■ Yes ☐ No
	2-7. Does it include the regulations for compensation and safety of research subjects?	■ Yes ☐ No
Tests	**3. Management of research subjects**	**Inspection result**
	3-1. Have all records on the identity of research subjects been handled in accordance with relevant regulations to ensure confidentiality??	■ Yes ☐ No
	3-2. Have all research subjects provided voluntary consent to participate in the clinical test in advance with a written signature?	■ Yes ☐ No
	3-3. Was consent to participate in the clinical test received through representatives?	☐ Yes ■ No
	3-4. If an adverse reaction occurred during the test, were appropriate measures taken according to the SOP?	☐ Yes ☐ No ■ N/A
	3-5. Were there any complaints from research subjects during the test?	☐ Yes ■ No

KDRI Korea Dermatology Research Institute

The contents of this report are confidential and cannot be used for other purposes unless approved.
All or any part of the contents in this report cannot be copied or published without consent of KDRI.

Tests	**4. Inspection during the main test**	**Inspection result**
	4-1. Was the test performed in the order of the planned steps according to the test plan and SOP?	■ Yes ☐ No
	4-2. Was the test performed using the measuring equipment and reagents planned according to the test plan and SOP?	■ Yes ☐ No
	4-3. Have the test equipment and facilities been safely stored and organized after completion of the test?	■ Yes ☐ No
	**5. Inspection during the result analysis step**	**Inspection result**
	5-1. Was data analysis conducted according to the test plan and SOP?	■ Yes ☐ No
	**6. Inspection of the final report**	**Inspection result**
	6-1. Has the result report been prepared according to the test plan and SOP?	■ Yes ☐ No
	6-2. Have the test plan, final report, basic test data, samples and documents related to the test been transferred to a designated storage facility?	■ Yes ☐ No
Documentation	**7. Do you keep the following basic documents? (Multiple selection possible)**<br>■ Test Plan<br>■Confirmation of Reliability Assurance<br>■ Case Report Form (CRF)<br>■ Contract<br>■ Affirmation Letter<br>■ Consent for Collection, Use, and Disclosure of Personal Information<br>■ Informed Consent<br>■ Initial Survey for Research Subjects<br>■ Compliance Log<br>■ Identification Numbers for Research Subjects<br>■ Description for Research Subjects<br>■ Compensation for Research Subjects<br>☐ Compensation Record<br>☐ Compensation Application and Confirmation<br>■ Double-blind Confirmation<br>■ CV of Tester(s)	

KDRI Korea Dermatology Research Institute

The contents of this report are confidential and cannot be used for other purposes unless approved.
All or any part of the contents in this report cannot be copied or published without consent of KDRI.

<table>
<tr><th colspan="6">Summary of Research Progress</th></tr>
<tr><th>Division</th><th colspan="4">No. of Research Subjects</th><th>Note</th></tr>
<tr><td>Planned No. of research subjects</td><td colspan="4">34</td><td>-</td></tr>
<tr><td>No. of Pre-screened research subjects</td><td colspan="4">34</td><td>-</td></tr>
<tr><td>No. of recruited research subjects</td><td colspan="4">34</td><td>-</td></tr>
<tr><td>No. of dropouts</td><td colspan="4">3</td><td>-</td></tr>
<tr><td>No. of final research subjects</td><td colspan="4">31</td><td>-</td></tr>
<tr><td rowspan="2">Test progress</td><td colspan="2">Test Start</td><td colspan="2">Test End</td><td rowspan="2">-</td></tr>
<tr><td colspan="2">Test group 17
Placebo group 17
(total 34)</td><td colspan="2">Test group 15
Placebo group 16
(total 31)</td></tr>
<tr><td rowspan="2">Reason of dropout</td><td>Withdrawal of consent</td><td>Non-compliance with schedule</td><td>Adverse reaction</td><td>Others*</td><td rowspan="2">-</td></tr>
<tr><td>0</td><td>3</td><td>0</td><td>0</td></tr>
</table>

Korea Dermatology Research Institute

Principal Investigator	Dermatology Specialist<br>**Lee, Kyungreal, MD**	(signature)
Research Director	**Lee, Donghwan**	(signature)
Director of Quality Assurance Duty	**Oh, Jongjin, MD**	(signature)

The contents of this report are confidential and cannot be used for other purposes unless approved.
All or any part of the contents in this report cannot be copied or published without consent of KDRI.

3. Background of Evaluation Test

Alopecia is a relatively common disease that begins in adults after adolescence. As the social interest in improving living standards and skin care increases, the interest in the treatment and prevention measures is also increasing. The causes of hair loss include genetic factors, poor eating habits, stress, seborrheic dandruff, endocrine abnormalities, etc. And the external application used for preventing hair loss, etc., is a functional cosmetic product, and the KFDA (Korean Food and Drug Administration) reviews and approves the safety and efficacy.

Androgen alopecia caused by man hormones and heredity occurs in about 50 percent of both men and women. It usually occurs in the 40s to 50s, but severe cases start shortly after puberty and last for decades. Both male and female hair loss begins mainly in calvaria and gradually progresses to the entire head. In the case of men, hair loss is predominant in calvaria and the receding of the headlines of both sides. In the case of a woman, the borderline of facial hair is generally well preserved, and the shape of a Christmas tree is common, and it is rare for a complete hair loss to be formed. In androgen alopecia, hair loss is DHT (Dihydrotestosterone) dependent, and DHT is produced by the 5AR (5-alpha-reductase) enzyme action from testosterone. 5AR has type 1 and type 2, of which type 2 is found in the papillary layer and outer roots of hair follicle, and it is presumed that it participates in androgenetic alopecia. Therefore, most hair loss treatment or scalp products with a hair having and hair growing effect often contain efficacy that has a 5AR inhibitory effect.

There are various methods to evaluate efficacy of hair saving or hair growth, from the invasive method such as biopsy to the non-invasive method such as phototrichogram, but the KFDA recommends the use of phototrichogram for hair loss-preventing functional cosmetics.

KDRI Korea Dermatology Research Institute

The contents of this report are confidential and cannot be used for other purposes unless approved.
All or any part of the contents in this report cannot be copied or published without consent of KDRI.

4. Purpose of Evaluation Test

This human body application test is a test to verify the effectiveness and safety of hair loss symptom alleviation through a human application test based on the principle of double blindness, placebo sample and randomization for 1 type of powder medicine (KDRI-2020-880-11-P) and 1 type of placebo (KDRI-2020-880-22-P) of functional cosmetics that help alleviate hair loss symptoms of the Leave on Type provided by the test requester.

The evaluation method follows the "Guidelines for the Effectiveness of Functional Cosmetics Used for Hair Loss Prevention, etc." issued by the Food and Drug Administration (Issued May 2017). Then, a visual evaluation of the researcher by questionnaire and photographing of the test subjects is conducted, and the validity is verified through phototrichogram.

KDRI Korea Dermatology Research Institute

The contents of this report are confidential and cannot be used for other purposes unless approved.
All or any part of the contents in this report cannot be copied or published without consent of KDRI.

4. Test Sample Identification

A. Sample Name and Physicochemical Properties

Sample Name	Sample Color and Form	Sample Code of KDRI
LEUCO-Exo (Sample 1) [Test sample]	White Powder	2020-880-11-P
Hair solvent (Sample 2) [Test sample]	Clear colorless liquid	2020-880-11-L
LEUCO-Exo (Sample 1) [Placebo sample]	White Powder	2020-880-22-P
Hair solvent (Sample 2) [Placebo sample]	Clear colorless liquid	2020-880-22-L

KDRI Korea Dermatology Research Institute

B. Components of Sample

Sample Name	Raw Materials Used in Manufacturing
LEUCO-Exo (Sample 1) [Test sample]	Leuconostoc extracellular vesicles, Mannitol
Hair solvent (Sample 2) [Test sample]	WATER, ALCOHOL DENAT., METHYLPROPANEDIOL, PANTHENOL, 1,2-HEXANEDIOL, CENTELLA ASIATICA EXTRACT, FICUS CARICA (FIG) FRUIT EXTRACT, HYDROGENATED LECITHIN, SODIUM HYALURONATE, SODIUM HYALURONATE, C12-14 PARETH-12, CARBOMER, CERAMIDE NP, COPPER TRIPEPTIDE-1, ETHYLHEXYLGLYCERIN, GLUTATHIONE, GLYCERIN, MENTHOL, MILK LIPIDS, NIACINAMIDE, SALICYLIC ACID, THEANINE, TROMETHAMINE
LEUCO-Exo (Sample 1) [Placebo sample]	Mannitol

The contents of this report are confidential and cannot be used for other purposes unless approved.
All or any part of the contents in this report cannot be copied or published without consent of KDRI.

Hair solvent (Sample 2)<br>[Placebo sample]	WATER, ALCOHOL DENAT., METHYLPROPANEDIOL, PANTHENOL, 1,2-HEXANEDIOL, CENTELLA ASIATICA EXTRACT, FICUS CARICA (FIG) FRUIT EXTRACT, HYDROGENATED LECITHIN, SODIUM HYALURONATE, SODIUM HYALURONATE, C12-14 PARETH-12, CARBOMER, CERAMIDE NP, COPPER TRIPEPTIDE-1, ETHYLHEXYLGLYCERIN, GLUTATHIONE, GLYCERIN, MENTHOL, MILK LIPIDS, NIACINAMIDE, SALICYLIC ACID, THEANINE, TROMETHAMINE

C. Active Ingredients of Test Sample

LACTOBACILLUS EXTRACELLULAR VESICLES

D. Sample Storage and Disposal

1) Store at temperatures between 1 ℃ and 30 ℃ avoiding both high temperatures and low temperatures, as well as direct sunlight.
2) Standard samples are stored in the lab for 1 month from the test end date, and are disposed of thereafter.
3) The evaluation samples provided to the subjects are collected after the completion of the test and discarded without further storing.

KDRI Korea Dermatology Research Institute

E. Sample Safety

1) The sample (s) was (were) manufactured under the Cosmetics Act.
2) The production of these samples did not use materials that could not be used in cosmetics in compliance with Ministry of Food and Drug Safety Notice 'Regulations on cosmetic safety standards, etc.', and 'Types of pigments, standard and test methods for cosmetics'. Also, materials requiring use limitations are produced in accordance with the prescribed limits of use and their standard for use.
3) The sample safety of these materials are guaranteed by the client.

The contents of this report are confidential and cannot be used for other purposes unless approved.
All or any part of the contents in this report cannot be copied or published without consent of KDRI.

6. Selection of Subjects

Among 18 to 54 years old adult men and women, those who meet the following selection criteria and who do not meet the exemption criteria are considered as subjects.

A. Selection Criteria

1) Male and female subjects diagnosed with androgenic hair loss between 18 and 54 years of age.
2) Male subjects diagnosed with 2 or more 2A or higher by the Norwood Hamiton classification, and female subjects diagnosed with 1 or more by the Ludwig classification.
3) A person who would not get any hair care or treatment during the test period.
4) A person who would maintain the same hair style during test period.
5) A person who was informed thoroughly about the test method, procedure and expected side effects & compensation & other matters regarding the test from test personnel, and voluntarily agreed on participation at the clinical test by signing the agreement.

KDRI Korea Dermatology Research Institute

B. Exemption Criteria

A person who corresponded to the followings through an interview was excluded from the subject.

1) A person who is suffering from acute severe kidney or heart disease, or other chronic disease (hypertension, diabetes, etc.), which could have affected the results of the test, during recent 6 months
2) A woman who is pregnant or breast feeding and possibly pregnant
3) A person with psychiatric disorders
4) A person with infectious skin diseases
5) A person who have received surgical treatment, such as hair transplantation, scalp reduction, for the purpose of curing hair loss.
6) A person who has a history of taking Dutasteride or Finasteride during recent 6 month.
7) A person who is applying hair grower, hair growth promoters, and hair restorer on scalp during recent 1 month.
8) A person who is taking steroids, cytotoxic agents, vasodilators, anti-hypertensive, antiepileptic, beta-receptor blockers, bronchodilators, diuretics, spironolactone, cimetidine,

The contents of this report are confidential and cannot be used for other purposes unless approved.
All or any part of the contents in this report cannot be copied or published without consent of KDRI.

diazoxide, cyclosporine, ketoconazole, during recent 1 month

9) A person who is applying topical steroids on scalp.
10) A person who is considered difficult to apply test substance due to severe seborrheic dermatitis, scalp psoriasis and scalp infections.
11) A person who has alopecia areata, telogen effluvium, cicatricial alopecia, except androgenetic alopecia.
12) A person who lack nutrition due to excessive dietary restrictions or gastrotomy or who take nutritional supplements that may affect hair loss
13) A person who is considered not suitable for the test by test personnel

C. Dropout Criteria

In the case of the subject's request or the reasons below, the subject will be dropped by the researcher's decision and but they are included in the safety assessment. The reason for dropping is recorded in detail in the CRF (Case Report Form).

1) In case of adverse reactions, such as flushing or red spots, on the test site during human application test
2) Serious skin adverse reaction after use of test sample
3) If the subject used drugs or physical procedures that could affect the hair loss symptoms outside the test specimen during the course of the test
4) If the subject is difficult to assess because of a violation of the method or schedule of use
5) If the subject wishes to stop the test
6) If the subject is unable to follow up due to personal reasons during the test
7) In the event that the study subjects cause a visually identified hairstyle and color change (such as a permanent wave or dyeing) during the test
8) If the other person in charge of the test judges that it is difficult to continue the test
9) The researcher directly contacts the test subjects using all methods such as phone calls, letters, and direct visits to subjects who refuse to visit to inform them of the adverse reaction.

KDRI Korea Dermatology Research Institute

D. Management of Subjects

The client and the test personnel of this research complied with the fundamental spirit of Helsinki Declaration to protect the rights and interests of the subjects, Good Clinical Practice (GCP) and related domestic regulations in executing and recording the research results.

The contents of this report are confidential and cannot be used for other purposes unless approved.
All or any part of the contents in this report cannot be copied or published without consent of KDRI.

The informed consent to test participation signed by all subjects were submitted before conducting the test and all the information necessary for the agreement according to [Guideline for cosmetic clinical study and efficacy test, 2015] published by Ministry of Food and Drug Safety of Korea (MFDS), were fully delivered to the subjects.

7. Test Method

A. Test Sample Application Area

Scalp

B. Test Sample Application Method

1) The study subjects self-use the appropriate amount of samples twice a week under the control and supervision of the tester. In order to make the test sample application identical to the actual usage of the product, subjects were educated on the application method(Self-application by subjects on the scalp two a week) specified by the client.
   (1) After opening the cap of the first LEUCO-Exo ampoule by tilting it back, remove the cap.
   (2) Remove the cap by tilting the cap of the second hair solvent ampoule back and opening it, and the enclosed exclusive use.
   (3) Cover the mixed ampoule and shake it until it melts completely.
   (4) After inhaling with the enclosed dedicated syringe, press with your finger to use an appropriate amount.
2) Double-blind and random allocation
   The test sample and the Placebo sample shall be distributed in the same container so that both the subject and the tester do not know the contents. Separate the test sample from the Placebo sample under the supervision of a researcher who do not participate in the test, and seal the sample code used to distinguish it so that it is not exposed.

KDRI Korea Dermatology Research Institute

B. Equipment Used

1) Folliscope® (Lead M, Korea)

In order to analyze and compare the characteristics of hair, the amount or type of hair per

The contents of this report are confidential and cannot be used for other purposes unless approved.
All or any part of the contents in this report cannot be copied or published without consent of KDRI.

unit area must be quantitatively measured for a sufficient amount of hair groups to obtain statistically meaningful results. For this purpose, Phototrichogram, which compares a photograph taken with a short cut of a certain area of hair and a photograph taken again of the same area after a certain period of time, can quantitatively measure non-invasively various indicators of hair, has less error in data, and is relatively easy to analyze. This study used a hair and scalp magnifying glass, Folliscope, in order to use these phototrichogram methods. Folliscope is a system device that can analyze the main characteristics of hair; that is, density, thickness, growth rate and gap between hairs, using a computer mounted with a high-resolution video microscopic camera and separate Folliscope software.

2) Digital camera (Canon EOS 750D) / Photograph shooting stand (Self-produced by KDRI)

Clinical photography is a device that can be attached to a photograph shooting stand with a digital camera (Canon EOS 750D), and can capture the vertex and hairline at the same angle and position.

KDRI Korea Dermatology Research Institute

C. Test Procedure

1) 2 weeks before start of the test

- The subjects of the study.
- Subjects were informed thoroughly about the test method, procedure, schedule, danger, potential adverse reactions, etc., filled out a form providing their basic personal information and signed an informed consent form.
- A one scalp micropigmentation is performed on scalps with hair loss.
  (Test area : A point about 15 cm away from the middle of the forehead, 3 cm to the left or right.)
- Offer the wash-out

2) Start of the test

- The photography of the hair and phototrichogram analysis was performed on the test area using Folliscope®
- Offer the sample.

The contents of this report are confidential and cannot be used for other purposes unless approved.
All or any part of the contents in this report cannot be copied or published without consent of KDRI.

3) 4 weeks start of the test

- The photography of the hair and phototrichogram analysis was performed on the test area using Folliscope®
- Visual and safety evaluation of 2 researchers by taking photos and Folliscope photographing
- Subjects completed a self-assessment questionnaire regarding their perceptions of hair condition.
- During the test period, subinvestigators checked whether the subjects used the test sample.

4) 8 weeks start of the test

- The photography of the hair and phototrichogram analysis was performed on the test area using Folliscope®
- Visual and safety evaluation of 2 researchers by taking photos and Folliscope photographing
- Subjects completed a self-assessment questionnaire regarding their perceptions of hair condition.
- During the test period, subinvestigators checked whether the subjects used the test sample.

KDRI Korea Dermatology Research Institute

5) 12 weeks start of the test

- The photography of the hair and phototrichogram analysis was performed on the test area using Folliscope®
- Visual and safety evaluation of 2 researchers by taking photos and Folliscope photographing
- Subjects completed a self-assessment questionnaire regarding their perceptions of hair condition.
- Return the sample.
- Pay the test participation fee.

D. Analysis of the data

1) Phototrichogram analysis using Folliscope®

- After cutting the hair at the test site, tattoo 1 mm in diameter is made and phototricogram is performed around the tattoo area.
- Hair density was measured as a number of hairs (N/cm^2) in the test area.

The contents of this report are confidential and cannot be used for other purposes unless approved.
All or any part of the contents in this report cannot be copied or published without consent of KDRI.

2) Self-assessment score by subjects

- Subjects completed a self-assessment questionnaire regarding their perceptions of hair condition at each visit (7 scale).
  ① Assessment of hair volume
  ② Assessment of degree of hair loss
  ③ Assessment of Change of hairline

Very improved	Improved	Little improved	No change	Little poor	Poor	Very poor
+3	+2	+1	0	-1	-2	-3

3) Evaluation of researchers by photographing

- Using a high-resolution digital camera (DSLR), take the human body photos at a designated location always under the same conditions, and then compare before and after applying the test samples.
- Human body photographing: The two areas of 45° (Bangs line) and 90° (Top of the head) are taken at the same conditions, angles, and location at each evaluation.
- Photograph evaluation: Visual evaluation is performed by 2 researchers on a 7-step scale, and the average of visual evaluation results of the 2 researchers is used as an effectiveness evaluation variable.
  Example > As a researcher, what do you judge the degree of hair distribution of the study subjects compared to before the test?

Very improved	Improved	Little improved	No change	Little poor	Poor	Very poor
+3	+2	+1	0	-1	-2	-3

4) Evaluation of skin irritancy by dermatologist

- At each visit, dermatologist evaluated any possible cutaneous adverse events such as
  ① Cutaneous adverse reactions by subjects: Pruritus, Stinging, Burning, Tingling, Stiffness, Others.
  ② Cutaneous adverse reactions by researchers: Erythema, Edema, Papule, Others.

The contents of this report are confidential and cannot be used for other purposes unless approved.
All or any part of the contents in this report cannot be copied or published without consent of KDRI.
Korea Dermatology Research Institute

5) Assessment of compliance

- After the end of the test, check the compliance of the sample as follows.

· Compliance(%)

= Number of times the actual sample was used / Number of times the sample should be used

6) Efficacy endpoint

A) Primary efficacy endpoint

① Phototrichogram - Hair density was measured as a number of hairs (N/cm^2)

B) Secondary efficacy endpoint

① Self-assessment score by subjects

② Visual evaluation score by researchers

7) Criteria for evaluating results

- It should show a statistically significant effect compared with the hair number control group in a circle with a size of 1cm^2 measured at the same position and it can be confirmed by the secondary efficacy evaluation variable there must be.

KDRI Korea Dermatology Research Institute

The contents of this report are confidential and cannot be used for other purposes unless approved.
All or any part of the contents in this report cannot be copied or published without consent of KDRI.

E. Statistical Analysis Method

Minitab 19 (Minitab® 19.2, Minitab Inc.) program is used to check the statistical significance.

- If the measured value of the test area is estimated as a normal distribution by Ryan-Joiner Normality Test, the statistical significance is confirmed by using the following parametric statistical methods:
- Comparisons between before and after measurements within the same group: paired t-test is used at the statistical significance level of $p<0.05$, and the statistical significance is confirmed by one-way ANOVA when repeated 3 or more times.
- Comparisons between more than 2 different groups: Welch's t-test is used at the statistical significance level of $p<0.05$ for the comparison between more than 2 different groups and one-way ANOVA is used at the statistical significance level of $p<0.05$ for the comparison between more than 3 different groups.
- Comparisons between more than 2 different groups calculated repeatedly: Repeated Measure ANOVA is used at the statistical significance level of $p<0.05$ and if the initial measurement value between the groups are different at statistical significance level, difference of the test value between the groups are calculated using Analysis of Covariance.
- When the normality is rejected, the significance is confirmed by the following non-parametric statistical method.
- Comparisons between before and after measurements within the same group: The Wilcoxon signed rank test was used to compare the values measured one time before and after each test, and the significance was confirmed by Kruskal-Wallis test when the test was repeated 3 times or more.
- Comparisons between more than 2 different groups: The Mann-Whitney U test was used to compare the measured values before and after each test. When the test was repeated 3 times or more, the significance was confirmed by Friedman test.
- Continuous variables are summarized by mean and standard deviation, and categorical variables by frequency and percentage.

KDRI Korea Dermatology Research Institute

The contents of this report are confidential and cannot be used for other purposes unless approved.
All or any part of the contents in this report cannot be copied or published without consent of KDRI.

8. Test Results

Table 1. Basic Information of Subjects

No.	ID	Age	Gender	type	No.	ID	Age	Gender	type
1	5182	25	Male	2A	18	2984	47	Female	Grade 1
2	4567	38	Male	2	19	5648	47	Female	Grade 1
3	2603	38	Female	Grade 1	20	3668*	48	Female	Grade 1
4	2793	40	Female	Grade 1	21	4005	49	Female	Grade 1
5	2884	40	Female	Grade 1	22	1719	49	Female	Grade 1
6	1984	41	Female	Grade 2	23	2909	49	Female	Grade 1
7	4014	42	Female	Grade 1	24	897	50	Female	Grade 1
8	3940	43	Female	Grade 1	25	3964	51	Female	Grade 1
9	2498	43	Female	Grade 2	26	4419*	51	Female	Grade 1
10	2379	44	Female	Grade 1	27	1297	52	Female	Grade 1
11	3238	44	Female	Grade 1	28	1482	52	Female	Grade 1
12	3822	44	Female	Grade 1	29	5646	52	Male	5
13	3943	45	Female	Grade 1	30	4303	53	Female	Grade 1
14	4167	45	Female	Grade 1	31	4997	53	Female	Grade 1
15	763	46	Female	Grade 1	32	4318	53	Male	2
16	2515	47	Female	Grade 1	33	4077	53	Female	Grade 1
17	4203*	47	Female	Grade 1	34	2824	53	Female	Grade 1

* Drop out

KDRI Korea Dermatology Research Institute

The contents of this report are confidential and cannot be used for other purposes unless approved.
All or any part of the contents in this report cannot be copied or published without consent of KDRI.

Table 2. Basic Information of Subjects - Summary

**Recruitment**	34	
**Drop out**	3	
**Progress**	31	
**Gender**	Male : 4	Female : 27
**Average Age**	46	
**Age Distribution**		
20s	1	
30s	2	
40s	18	
50s	10	

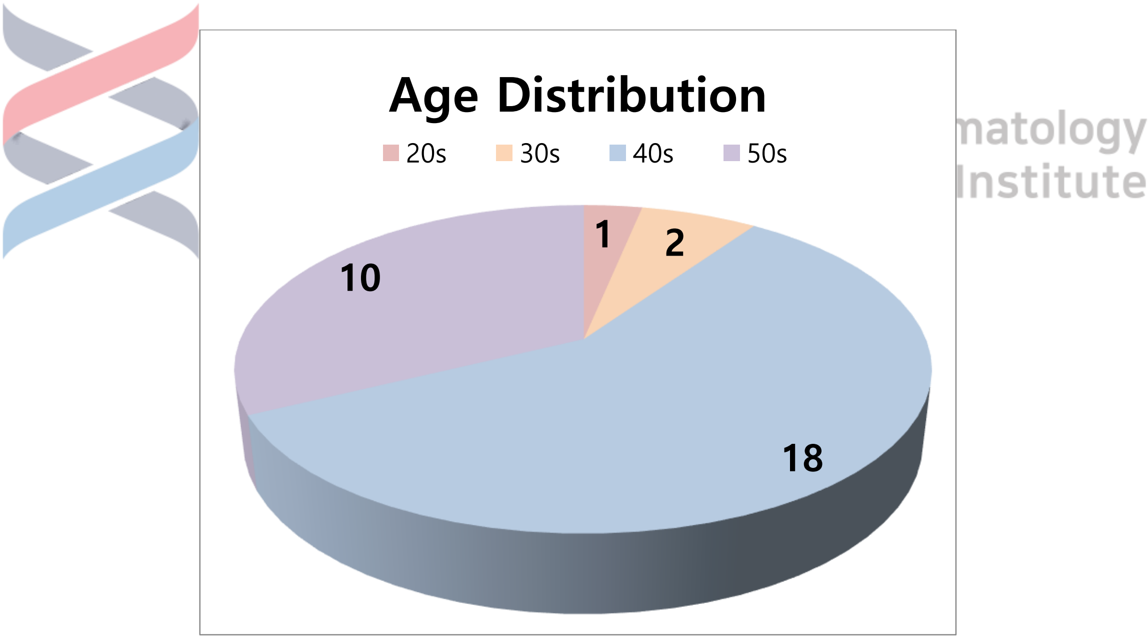

Fig 1. Age Distribution of the study subjects

Table 3. Progress of test

Week 4	Week 8	Week 12
Test group 15<br>Placebo group 16<br>(Total 31)	Test group 15<br>Placebo group 16<br>(Total 31)	Test group 15<br>Placebo group 16<br>(Total 31)

The contents of this report are confidential and cannot be used for other purposes unless approved.
All or any part of the contents in this report cannot be copied or published without consent of KDRI.

Table 4. Hair density measuring result (N/cm²)

No.	Test Group				Placebo Group			
	Week 0	Week4	Week 8	Week 12	Week 0	Week4	Week 8	Week 12
1	120	128	134	134	90	80	88	94
2	137	143	146	152	112	112	110	107
3	94	109	110	121	142	145	142	148
4	93	105	104	104	128	128	128	128
5	110	124	128	120	107	112	105	112
6	121	123	124	123	109	113	93	107
7	112	118	121	128	118	116	121	118
8	93	104	102	105	121	121	121	124
9	-	-	-	-	115	113	115	107
10	112	-	-	-	118	120	119	123
11	91	101	108	112	109	116	105	104
12	79	91	94	90	96	-	-	-
13	115	121	123	127	147	147	147	148
14	112	113	113	115	80	79	77	79
15	86	104	105	105	115	113	117	115
16	130	132	135	142	90	88	90	88
17	107	123	134	145	113	112	112	104

KDRI Korea Dermatology Research Institute

The contents of this report are confidential and cannot be used for other purposes unless approved.
All or any part of the contents in this report cannot be copied or published without consent of KDRI.

Table 5. Variation for Hair density measuring result (N/cm²)

No.	Test Group			Placebo Group		
	Week4	Week 8	Week 12	Week4	Week 8	Week 12
1	8	14	14	-10	-2	4
2	6	9	15	0	-2	-5
3	15	16	27	3	0	6
4	12	11	11	0	0	0
5	14	18	10	5	-2	5
6	2	3	2	4	-16	-2
7	6	9	16	-2	3	0
8	11	9	12	0	0	3
9	-	-	-	-2	0	-8
10	-	-	-	2	1	5
11	10	17	21	7	-4	-5
12	12	15	11	-	-	-
13	6	8	12	0	0	1
14	1	1	3	-1	-3	-1
15	18	19	19	-2	2	0
16	2	5	12	-2	0	-2
17	16	27	38	-1	-1	-9

The contents of this report are confidential and cannot be used for other purposes unless approved.
All or any part of the contents in this report cannot be copied or published without consent of KDRI.

Table 6. Visual evaluation result by researcher 1

(3: very improved, 2: improved, 1: little improved, 0: no change, -1: little poor, -2: poor, -3: very poor)

No.	Test Group			Placebo Group		
	Week4	Week 8	Week 12	Week4	Week 8	Week 12
1	0	1	1	0	0	0
2	0	0	0	0	0	0
3	0	0	1	0	0	0
4	0	0	0	0	0	0
5	0	0	0	0	0	0
6	0	0	0	0	0	0
7	0	1	1	0	0	0
8	0	0	0	0	0	0
9	-	-	-	0	0	0
10	-	-	-	0	0	0
11	0	0	1	0	0	0
12	0	0	0	-	-	-
13	0	0	0	0	0	0
14	0	0	0	0	-1	-1
15	0	0	0	0	0	0
16	0	0	0	0	-1	-1
17	0	0	0	0	0	0

KDRI Korea Dermatology Research Institute

The contents of this report are confidential and cannot be used for other purposes unless approved.
All or any part of the contents in this report cannot be copied or published without consent of KDRI.

Table 7. Visual evaluation result by researcher 2

(3: very improved, 2: improved, 1: little improved, 0: no change, -1: little poor, -2: poor, -3: very poor)

No.	Test Group			Placebo Group		
	Week4	Week 8	Week 12	Week4	Week 8	Week 12
1	0	1	1	0	0	0
2	0	0	0	0	0	0
3	0	0	1	0	0	0
4	1	0	1	-1	0	0
5	0	0	0	0	0	0
6	0	0	0	1	0	1
7	0	1	1	0	0	0
8	0	0	0	0	0	0
9	-	-	-	0	0	0
10	-	-	-	0	0	0
11	0	0	1	0	0	0
12	0	1	1	-	-	-
13	0	0	0	0	0	0
14	0	0	0	0	-1	-1
15	0	0	0	0	0	0
16	0	1	-2	0	-1	-1
17	0	0	0	0	0	0

Korea Dermatology Research Institute

The contents of this report are confidential and cannot be used for other purposes unless approved.
All or any part of the contents in this report cannot be copied or published without consent of KDRI.

Table 8. Visual evaluation final result by researchers

(3: very improved, 2: improved, 1: little improved, 0: no change, -1: little poor, -2: poor, -3: very poor)

No.	Test Group			Placebo Group		
	Week4	Week 8	Week 12	Week4	Week 8	Week 12
1	0	1	1	0	0	0
2	0	0	0	0	0	0
3	0	0	1	0	0	0
4	0	0	0	-1	0	0
5	0	0	0	0	0	0
6	0	0	0	0	0	0
7	0	1	1	0	0	0
8	0	0	0	0	0	0
9	-	-	-	0	0	0
10	-	-	-	0	0	0
11	0	0	1	0	0	0
12	0	0	0	-	-	-
13	0	0	0	0	0	0
14	0	0	0	0	-1	-1
15	0	0	0	0	0	0
16	0	0	-2	0	-1	-1
17	0	0	0	0	0	0

KDRI Korea Dermatology Research Institute

The contents of this report are confidential and cannot be used for other purposes unless approved.
All or any part of the contents in this report cannot be copied or published without consent of KDRI.

Table 9. Self-assessment score by subjects – Hair volume

(3: very improved, 2: improved, 1: little improved, 0: no change, -1: little poor, -2: poor, -3: very poor)

No.	Test Group			Placebo Group		
	Week4	Week 8	Week 12	Week4	Week 8	Week 12
1	1	1	1	0	1	1
2	1	1	1	0	1	1
3	0	1	2	0	0	0
4	2	1	1	0	1	1
5	1	1	1	1	1	1
6	3	3	3	0	0	0
7	1	1	1	1	1	2
8	0	1	1	0	0	0
9	-	-	-	0	1	1
10	-	-	-	0	1	1
11	3	3	3	-1	-1	-1
12	0	0	1	-	-	-
13	1	2	1	0	0	0
14	0	0	0	0	0	0
15	1	1	1	1	0	0
16	1	1	1	1	1	1
17	2	3	3	0	1	1

KDRI Korea Dermatology Research Institute

The contents of this report are confidential and cannot be used for other purposes unless approved.
All or any part of the contents in this report cannot be copied or published without consent of KDRI.

Table 10. Self-assessment score by subjects – Degree of hair loss

(3: very improved, 2: improved, 1: little improved, 0: no change, -1: little poor, -2: poor, -3: very poor)

No.	Test Group			Placebo Group		
	Week4	Week 8	Week 12	Week4	Week 8	Week 12
1	2	1	1	1	1	1
2	1	1	1	1	1	1
3	1	1	2	0	0	0
4	2	1	1	0	0	0
5	2	1	1	1	1	1
6	3	2	2	-1	-1	-2
7	1	0	0	1	2	2
8	1	1	1	0	1	1
9	-	-	-	1	1	1
10	-	-	-	0	1	1
11	3	3	3	-1	-1	-1
12	0	0	1	-	-	-
13	2	2	1	1	1	1
14	0	1	1	0	0	0
15	1	2	1	1	0	0
16	1	1	1	0	0	1
17	1	1	1	1	1	1

KDRI Korea Dermatology Research Institute

The contents of this report are confidential and cannot be used for other purposes unless approved.
All or any part of the contents in this report cannot be copied or published without consent of KDRI.

Table 11. Self-assessment score by subjects – Change of hairline

(3: very improved, 2: improved, 1: little improved, 0: no change, -1: little poor, -2: poor, -3: very poor)

No.	Test Group			Placebo Group		
	Week4	Week 8	Week 12	Week4	Week 8	Week 12
1	0	1	1	0	0	0
2	0	2	2	0	1	1
3	1	1	2	0	0	0
4	1	1	2	0	1	1
5	1	0	1	0	0	0
6	3	3	2	-1	-1	-1
7	1	1	1	0	1	2
8	1	1	1	0	0	0
9	-	-	-	0	1	1
10	-	-	-	0	1	1
11	3	3	3	-1	-1	-1
12	1	1	2	-	-	-
13	1	2	2	0	0	0
14	0	1	1	0	0	0
15	0	2	1	1	0	0
16	1	2	2	0	1	1
17	0	0	1	0	0	1

KDRI Korea Dermatology Research Institute

The contents of this report are confidential and cannot be used for other purposes unless approved.
All or any part of the contents in this report cannot be copied or published without consent of KDRI.

A. Primary efficacy endpoint - Hair density (N/cm²)

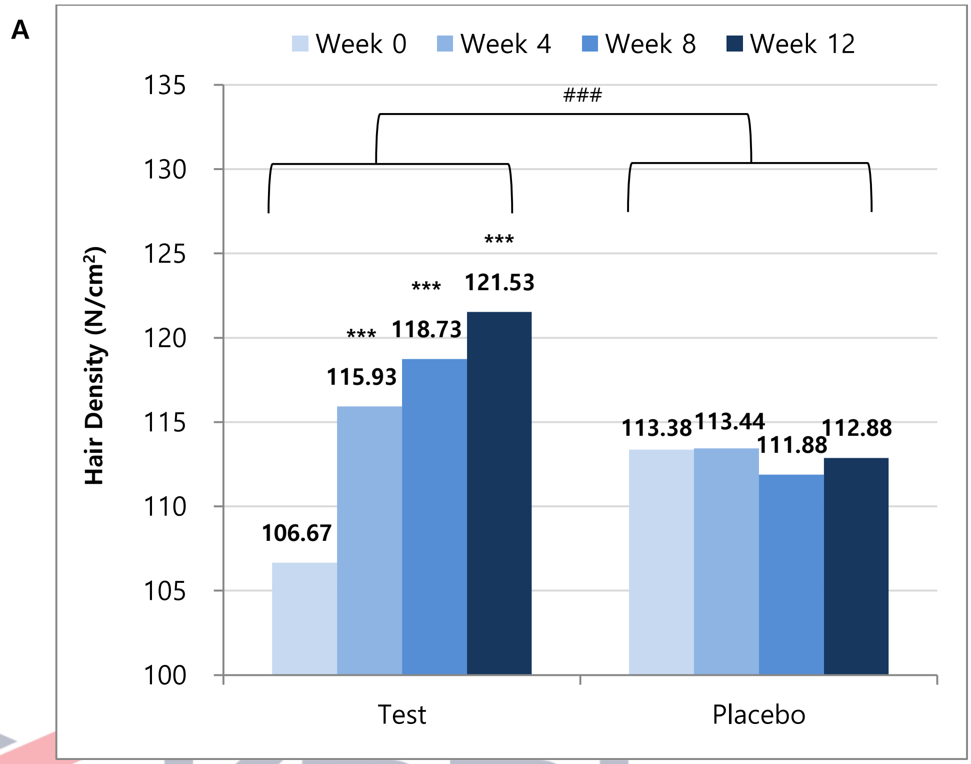

*Significant probability within group: *p<0.05, **p<0.01, ***p<0.001*
Significant probability between group in interaction: #p<0.05, ##p<0.01, ###p<0.001

KDRI Korea Dermatology Research Institute

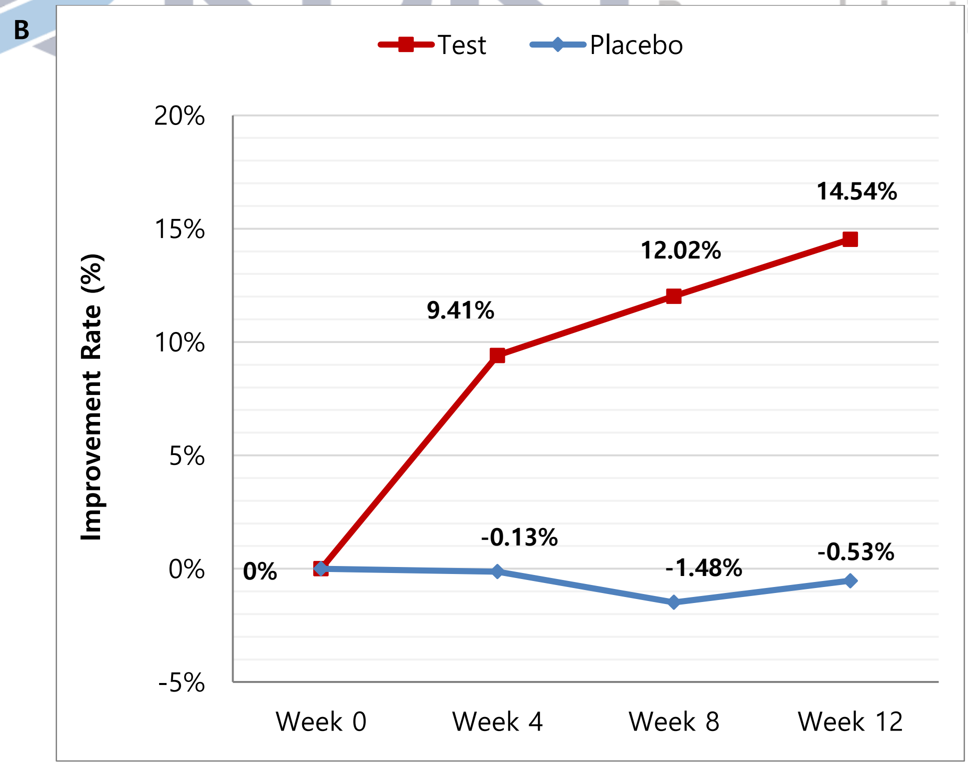

Fig 2. Change of Hair Density (N/cm²) (A: Hair Density, B: Improvement Rate)

The contents of this report are confidential and cannot be used for other purposes unless approved.
All or any part of the contents in this report cannot be copied or published without consent of KDRI.

Table 12. Homogeneity Statistical Result of Hair Density(N/cm^2)

Division	Mean ± S.D.		*p*-value (Between Group)
	Test	Placebo	
Week 0	106.67 ± 16.81	113.38 ± 17.43	**0.284** [1)]

1) by Welch's t-test.

As a result of checking the normality for the measurement results of the number of hair (N/cm^2), the normality of all measurement results could be estimated. Furthermore, before the test starts, as a result of checking the homogeneity of hair number of the study subjects in the test and the placebo groups, it was confirmed that the number of hair between the two groups did not have a statistically significant level difference ($p<0.05$).

Therefore, the comparison before and after the test and between the groups for the measurement results of the number of hair in the test and placebo groups was parametricly analyzed through Repeated Measure ANOVA.

Table 13. Statistics Result of Hair Density(N/cm^2)

Division		Mean ± S.D.	Improvement Rate	*p*-value (Within Group)	*p*-value (Between Group)
Test	Week 0	106.67 ± 16.81 [a]	-	**<0.001** [1)]	**0.645** [2)] **<0.001** [3)]
	Week 4	115.93 ± 13.72 [b]	9.41%		
	Week 8	118.73 ± 14.96 [bc]	12.02%		
	Week 12	121.53 ± 17.14 [c]	14.54%		
Placebo	Week 0	113.38 ± 17.43 [a]	-	**0.615** [1)]	
	Week 4	113.44 ± 18.98 [a]	-0.13%		
	Week 8	111.88 ± 18.93 [a]	-1.48%		
	Week 12	112.88 ± 18.86 [a]	-0.53%		

abc : Averages that do not share characters have a statistically significant difference ($p<0.05$). by Tukey test.
1) by Repeated measures ANOVA. Analyze the time of measurement as a factor.
2) by Repeated measures ANOVA. Analyze groups as factors.
3) by Repeated measures ANOVA. Analyze interaction (Group*Week) as a factor.

The contents of this report are confidential and cannot be used for other purposes unless approved.
All or any part of the contents in this report cannot be copied or published without consent of KDRI.

As a result of analyzing the degree of change and before and after the test of the measurement results of the number of hair (N/cm^2), in the case of the test group, the number of hair increased to a statistically significant level (p<0.05) from the 4th week compared to before the test, and in the case of the placebo group, the change in the number of hair could not be identified at a statistically significant level (p<0.05). These results were also able to confirm the significance through a Post-Hoc test.

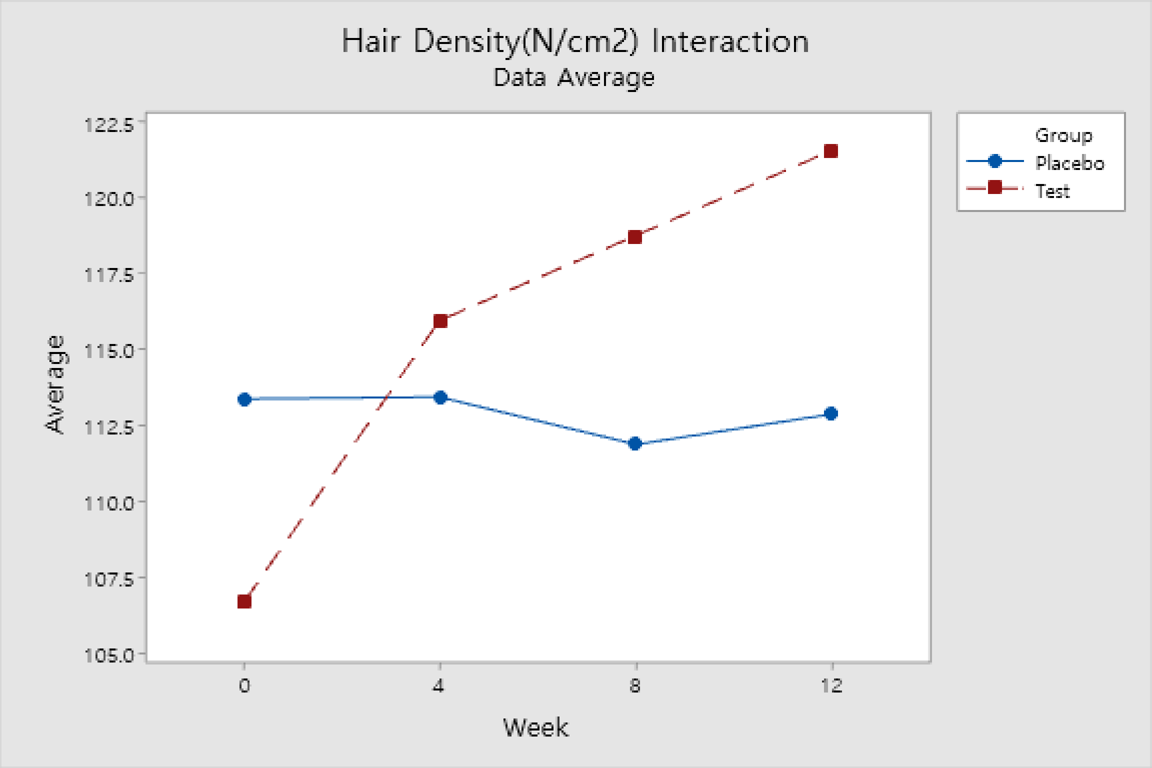

Fig 3. Hair Density Interaction (Group x Week, Minitab® 19.2, Minitab Inc.)

In order to see the difference in the number of hairs between the groups, a repeated measures ANOVA was performed using two factors. In the first analysis, the difference was analyzed by correcting the measurement time in order to exclude the change in the number of hairs according to the measurement time with the groups as a factor.

The second analysis was performed on interaction, meaning that the effect of one factor (groups) depends on the level of the other factor (the number of weeks, test period). In this study, the statistical analysis explains changes according to the time of measurement. If the level of interaction of each group is shown as parallel lines, there is no interaction. However, the greater the difference in the inclination of the lines, the greater the interaction. Therefore, an error of interpretation, which was made by the difference between the groups in the group factor before the test, can be

The contents of this report are confidential and cannot be used for other purposes unless approved.
All or any part of the contents in this report cannot be copied or published without consent of KDRI.

supplemented with the interpretation of the change pattern through interaction. Previous studies that obtained the results by evaluating interaction through time-dependent repeated measurements of longitudinal data, just like this test, include research by Kramer et al. and Holthoff et al.[A),B)] Research performed by Andersson et al.[C)] provided a detailed description of the effects of interactions.

As a result of the analysis, no statistically significant difference (p<0.05) was found in the results when comparing the number of hairs between the groups for the entire test period, including before using the sample.

This is due to the homogeneity between the groups being strong when just started to use the sample, while more differences occurred between the test and placebo groups as they used it for a longer period. When the difference was not significant, or at the beginning of using the sample, the placebo group showed dominant measurements, while the trend lines of the groups crossed each other as they use it for a longer period; this is expected to be a result of the test group measurements becoming dominant.

An additional interaction analysis showed a statistically significant difference (p<0.05), revealing that there was a difference in the pattern of changes between the groups according to the period of sample use. This can be supported by the results of a comparative analysis of the number of hairs measured at different time points (△) between the groups.

KDRI Korea Dermatology Research Institute

Table 14. Statistics Result of Hair Density(N/cm^2) Variation(△) by Period of use

Division	Mean ± S.D.		*p*-value (Between Group)
	Test	Placebo	
4 Week Variation(△)	9.27 ± 5.36	0.06 ± 3.84	**<0.001** 1)
8 Week Variation(△)	12.07 ± 6.86	-1.50 ± 4.26	**<0.001** 2)
12 Week Variation(△)	14.87 ± 8.96	-0.50 ± 4.56	**<0.001** 1)

1) by Welch's t-test.

2) by Mann-whitney u test.

In order verify the statistical significance between the test and the control groups of the variation (△) in the measurement value of the number of hair by period, as a result of additional statistical analysis between the groups, in all 4 week, 8 week, and 12 week, it was confirmed that the variation between the test and the placebo groups had a statistically significant level difference (p<0.017).

The contents of this report are confidential and cannot be used for other purposes unless approved.
All or any part of the contents in this report cannot be copied or published without consent of KDRI.

B. Secondary efficacy endpoint - Visual evaluation score by researchers

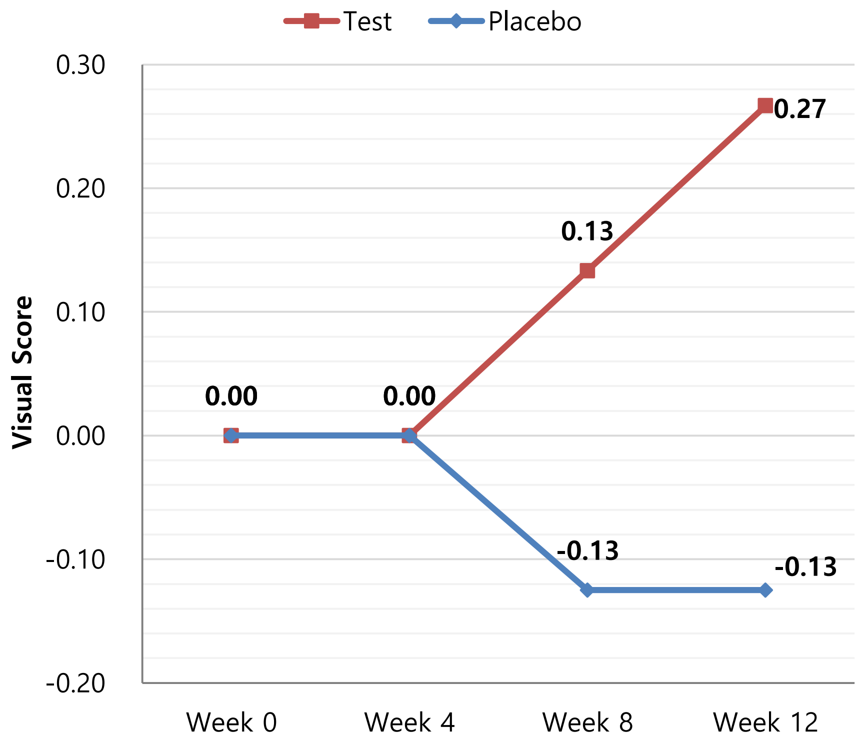

Fig 4. Change of Visual evaluation score by researchers

Korea Dermatology Research Institute

The contents of this report are confidential and cannot be used for other purposes unless approved.
All or any part of the contents in this report cannot be copied or published without consent of KDRI.

Table 15. Correlation Consistency Evaluation within The Grade between Visual Evaluation of Alopecia by Testers

Division	Number of Inspected Observations	Number of Matched Observations	Match Percentage (%)	Kendall's Consistency Coefficients		
				DF	Chi-square	Consistency Coefficients
Consistency between the Testers	93	84	90.32	92	155.74	0.85

* Consistency Coefficients ≥ 0.8: visual evaluation result match between two testers

The results of visual evaluation between the two testers were verified through the Intraclass Correlation Consistency Evaluation, showing a consistency of 0.8 or higher.

Table 16. Statistics Result of Visual Evaluation Score by Researchers

Division		Mean ± S.D.	*p*-value (Within Group)	*p*-value (Between Group)
Test	Week 4	0.00 ± 0.00 [a]	**0.247** [1)]	**0.064** [3)] **0.451** [4)]
	Week 8	0.13 ± 0.35 [a]		
	Week 12	0.13 ± 0.74 [a]		
Placebo	Week 4	-0.06 ± 0.25 [b]	**0.729** [2)]	
	Week 8	-0.13 ± 0.34 [b]		
	Week 12	-0.13 ± 0.34 [b]		

Korea Dermatology Research Institute

a / b : Averages that do not share characters have a statistically significant difference (p<0.05). by Bonferroni method / by Tukey test.

1) by Friedman test. Analyze the time of measurement as a factor.

2) by Repeated measures ANOVA. Analyze the time of measurement as a factor.

3) by Repeated measures ANOVA. Analyze groups as factors.

4) by Repeated measures ANOVA. Analyze interaction (Group*Week) as a factor.

As a result of examining the visual inspection results, normality was estimated in the placebo group, but not in the test group. Therefore, the comparison between before and after the test in the test group was analyzed non-parametrically by using a Friedman test, and a parametrical repeated measures ANOVA was used for the placebo group. After analyzing the changes according

The contents of this report are confidential and cannot be used for other purposes unless approved.
All or any part of the contents in this report cannot be copied or published without consent of KDRI.

to the period of sample use, a post-test was performed for additional comparison by week. Based on the normality test results, the test group was analyzed using a Bonferroni test and the placebo group using a Tukey test. As a result, neither the test group nor the placebo group showed a statistically significant change ($p<0.05$). In addition, a repeated measures ANOVA performed to investigate differences in the visual inspection of hair improvement between the two groups showed no statistically significant difference ($p<0.05$).

Table 17. Statistics Result of Visual Evaluation Score by Researchers by Period of use

Division	Mean ± S.D.		*p*-value (Between Group)
	Test	Placebo	
Week 4	0.00 ± 0.00	-0.06 ± 0.25	**N/A**
Week 8	0.13 ± 0.35	-0.13 ± 0.34	**0.047** [1)]
Week 12	0.13 ± 0.74	-0.13 ± 0.34	**0.233** [1)]

1) by Welch's t-test.

KDRI Korea Dermatology Research Institute

As a result of the additional statistical analysis to examine the statistical significance of the visual inspection of hair improvement between the groups at each time point, no statistically significant difference was found at an adjusted significance level ($p<0.017$).

The contents of this report are confidential and cannot be used for other purposes unless approved.
All or any part of the contents in this report cannot be copied or published without consent of KDRI.

C. Secondary efficacy endpoint - Self-assessment score by subjects

Table 18. Average self-assessment score –total

Evaluation Category		Test			Placebo		
		Week 8	Week 16	Week 24	Week 8	Week 16	Week 24
Hair volume	Very improved	2(13.3%)	3(20.0%)	3(20.0%)	0(0.0%)	0(0.0%)	0(0.0%)
	Improved	2(13.3%)	1(6.7%)	1(6.7%)	0(0.0%)	0(0.0%)	1(6.3%)
	Little improved	7(46.7%)	9(60.0%)	10(66.7%)	4(25.0%)	9(56.3%)	8(50.0%)
	No change	4(26.7%)	2(13.3%)	1(6.7%)	11(68.8%)	6(37.5%)	6(37.5%)
	Little poor	0(0.0%)	0(0.0%)	0(0.0%)	1(6.3%)	1(6.3%)	1(6.3%)
	Poor	0(0.0%)	0(0.0%)	0(0.0%)	0(0.0%)	0(0.0%)	0(0.0%)
	Very poor	0(0.0%)	0(0.0%)	0(0.0%)	0(0.0%)	0(0.0%)	0(0.0%)
**Mean**		**1.13**	**1.40**	**0.93**	**0.19**	**0.38**	**-0.06**
**S.D.**		**0.99**	**0.91**	**0.96**	**0.54**	**0.72**	**0.44**
Degree of hair loss	Very improved	2(13.3%)	1(6.7%)	1(6.7%)	0(0.0%)	0(0.0%)	0(0.0%)
	Improved	4(26.7%)	3(20.0%)	2(13.3%)	0(0.0%)	1(6.3%)	1(6.3%)
	Little improved	7(46.7%)	9(60.0%)	11(73.3%)	8(50.0%)	8(50.0%)	9(56.3%)
	No change	2(13.3%)	2(13.3%)	1(6.7%)	6(37.5%)	5(31.3%)	4(25.0%)
	Little poor	0(0.0%)	0(0.0%)	0(0.0%)	2(12.5%)	2(12.5%)	1(6.3%)
	Poor	0(0.0%)	0(0.0%)	0(0.0%)	0(0.0%)	0(0.0%)	1(6.3%)
	Very poor	0(0.0%)	0(0.0%)	0(0.0%)	0(0.0%)	0(0.0%)	0(0.0%)
**Mean**		**1.33**	**1.20**	**1.40**	**0.50**	**0.50**	**0.25**
**S.D.**		**0.98**	**0.77**	**0.91**	**0.63**	**0.82**	**0.68**
Change of hairline	Very improved	2(13.3%)	2(13.3%)	1(6.7%)	0(0.0%)	0(0.0%)	0(0.0%)
	Improved	0(0.0%)	4(26.7%)	7(46.7%)	0(0.0%)	0(0.0%)	1(6.3%)
	Little improved	8(53.3%)	7(46.7%)	7(46.7%)	1(6.3%)	6(37.5%)	6(37.5%)
	No change	5(33.3%)	2(13.3%)	0(0.0%)	13(81.3%)	8(50.0%)	7(43.8%)
	Little poor	0(0.0%)	0(0.0%)	0(0.0%)	2(12.5%)	2(12.5%)	2(12.5%)
	Poor	0(0.0%)	0(0.0%)	0(0.0%)	0(0.0%)	0(0.0%)	0(0.0%)
	Very poor	0(0.0%)	0(0.0%)	0(0.0%)	0(0.0%)	0(0.0%)	0(0.0%)
**Mean**		**1.40**	**1.20**	**1.60**	**0.56**	**0.50**	**0.38**
**S.D.**		**0.91**	**0.68**	**0.63**	**0.73**	**0.97**	**0.81**

KDRI Korea Dermatology Research Institute

The contents of this report are confidential and cannot be used for other purposes unless approved.
All or any part of the contents in this report cannot be copied or published without consent of KDRI.

Table 19. Statistics Result of Self-Assessment Score

Division			Mean ± S.D.	*p*-value (Within Group)	*p*-value (Between Group)
Hair volume	Test	Week 4	1.13 ± 0.99 [a]	**0.201** [1)]	**0.003** [2)] **0.813** [3)]
		Week 8	1.33 ± 0.98 [a]		
		Week 12	1.40 ± 0.91 [a]		
	Placebo	Week 4	0.19 ± 0.54 [a]	**0.016** [1)]	
		Week 8	0.50 ± 0.63 [ab]		
		Week 12	0.56 ± 0.73 [b]		
Degree of hair loss	Test	Week 4	1.40 ± 0.91 [a]	**0.421** [1)]	**0.005** [2)] **0.218** [3)]
		Week 8	1.20 ± 0.77 [a]		
		Week 12	1.20 ± 0.68 [a]		
	Placebo	Week 4	0.38 ± 0.72 [a]	**0.528** [1)]	
		Week 8	0.50 ± 0.82 [a]		
		Week 12	0.50 ± 0.97 [a]		
Change of hairline	Test	Week 4	0.93 ± 0.96 [a]	**0.006** [1)]	**<0.001** [2)] **0.623** [3)]
		Week 8	1.40 ± 0.91 [ab]		
		Week 12	1.60 ± 0.63 [b]		
	Placebo	Week 4	-0.06 ± 0.44 [a]	**0.015** [1)]	
		Week 8	0.25 ± 0.68 [ab]		
		Week 12	0.38 ± 0.81 [b]		

ab : Averages that do not share characters have a statistically significant difference ($p<0.05$). by Tukey test.

1) by Repeated measures ANOVA. Analyze the time of measurement as a factor.

2) by Repeated measures ANOVA. Analyze groups as factors.

3) by Repeated measures ANOVA. Analyze interaction (Group*Week) as a factor.

Korea Dermatology Research Institute

The contents of this report are confidential and cannot be used for other purposes unless approved.
All or any part of the contents in this report cannot be copied or published without consent of KDRI.

A repeated measures ANOVA examined changes in the hair improvement scores based on a survey with the research subjects using criteria including crown hair volume, hair shedding, and forelock improvement. A post-test was also conducted for additional comparison by week according to the normality test results using the Tukey test. As a result, no statistically significant change (p<0.05) was found in the test group with the criteria of crown hair volume, while it showed an increasing trend. On the other hand, the placebo group showed a statistically significant increase (p<0.05) after Week 12 compared to Week 8. As for hair shedding, both groups did not have a statistically significant change (p<0.05). For forelock improvement, both groups had a statistically significant increase (p<0.05) after Week 12 compared to Week 8. In addition, as a result of a repeated measures ANOVA conducted to examine the trend of improvement scores between the groups based on the survey with the research subjects, the criteria of hair volume, hair shedding, and forelock improvement showed statistically significant differences (p<0.05) between the test and placebo groups.

Table 20. Comparison Statistical Analysis of Self-Assessment Score of Test and Placebo group

Division		Mean ± S.D.		*p*-value (Between Group)
		Test	Placebo	
Hair volume	Week 4	1.13 ± 0.99	0.19 ± 0.54	**0.004** 1)
	Week 8	1.33 ± 0.98	0.50 ± 0.63	**0.010** 1)
	Week 12	1.40 ± 0.91	0.56 ± 0.73	**0.009** 1)
Degree of hair loss	Week 4	1.40 ± 0.91	0.38 ± 0.72	**0.002** 1)
	Week 8	1.20 ± 0.77	0.50 ± 0.82	**0.021** 1)
	Week 12	1.20 ± 0.68	0.50 ± 0.97	**0.026** 1)
Change of hairline	Week 4	0.93 ± 0.96	-0.06 ± 0.44	**0.002** 1)
	Week 8	1.40 ± 0.91	0.25 ± 0.68	**0.001** 1)
	Week 12	1.60 ± 0.63	0.38 ± 0.81	**<0.001** 1)

1) by Welch's t-test.

An additional analysis performed to examine the statistical significance of the evaluation scores by time point between the test and placebo groups revealed adjusted significant differences

The contents of this report are confidential and cannot be used for other purposes unless approved.
All or any part of the contents in this report cannot be copied or published without consent of KDRI.

($p<0.017$) in hair volume at Weeks 4, 8, and 12, hair shedding at Week 4, and forelock improvement at Weeks 4, 8, and 12. In addition, a pattern of increase was found in the hair volume and forelock improvement scores.

D. In use safety

Table 21. Self-Assessment of Irritation

Division	Pruritus	Stinging	Burning	Tingling	Stiffness	Others
**Mild**	0	0	0	0	0	0
**Moderate**	0	0	0	0	0	0
**Severe**	0	0	0	0	0	0

No adverse reactions were observed.

KDRI Korea Dermatology

Table 22. Evaluation of Cutaneous Adverse Reactions

Division	Erythema	Edema	Papule	Others
**Mild**	0	0	0	0
**Moderate**	0	0	0	0
**Severe**	0	0	0	0

No adverse reactions were observed.

E. Compliance

Table 23. Compliance

Number of Subjects	Compliance (%)
31	99.87%

The contents of this report are confidential and cannot be used for other purposes unless approved.
All or any part of the contents in this report cannot be copied or published without consent of KDRI.

9. Discussion and Conclusions

Double blind, placebo controlled, single site, randomized, applied to the human body to evaluate the safety of samples and effectiveness of hair loss prevention for 34 subjects during the entire 12 week study period a test was conducted to secure 34 or more subjects and 31 final subjects completed the test except for 6 drop out subject. The test was conducted using a double-blind method, making it impossible to know the contents of the notated sample until both the tester and the subject A and B until the seal deletion until the end of the test. Evaluation was conducted by visual evaluation score by researcher, self-assessment score by subject and evaluation of equipment using Folliscope.

1) Primary efficacy endpoint - Hair density (N/cm^2)

   As a result of analyzing the initial homogeneity of the number of hairs of both groups, no statistically significant difference (p<0.05) was found, indicating that the test and placebo groups were homogeneous. As a result of analyzing changes in the number of hairs (N/cm^2) before and after the test through a phototrichogram, the test group showed a statistically significant increase (p<0.05) after Week 4, while the placebo group showed no statistically significant change (p<0.05). An interaction analysis of weekly changes in the number of hairs of the groups showed a statistically significant difference (p<0.05), and changes in the number of hairs in both groups showed statistically significant differences at an adjusted level (p<0.017) at Weeks 4, 8, and 12.

2) Secondary efficacy endpoint (1) - Visual evaluation score by researchers

   As a result of analyzing changes in the visual evaluation scores before and after the test, both groups did not show a statistically significant difference (p<0.05). However, the numbers revealed that the test group experienced improvement while the other group showed deterioration. No statistically significant difference (p<0.05) was found in the tendency between the two groups.

The contents of this report are confidential and cannot be used for other purposes unless approved.
All or any part of the contents in this report cannot be copied or published without consent of KDRI.

KDRI Korea Dermatology Research Institute

3) Secondary efficacy endpoint (2) - Self-assessment score by subjects

   The survey with the research subjects evaluated the improvement of crown hair volume, hair shedding, and forelock. The results found that the test group experienced improvement in forelock at a statistically significant level (p<0.05) after Week 12 compared to Week 8; the placebo group showed improvement in hair volume and forelock. The statistical analysis by time point also found statistically significant differences (p<0.05) between the groups in hair volume and forelock improvement at Weeks 4, 8, and 12, and hair shedding at Week 4.

4) During the test period, all subjects showed more than 90% compliance.

5) In addition, no special adverse reaction was observed during the testing period, therefore the sample is considered to be safety.

In conclusion, the test areas where **GFC Life Science Co.,Ltd. "LEUCO-Exo (Sample 1) & Hair solvent (Sample 2)"** were applied for 12 weeks showed alopecia prevention efficacy at a statistically significant level (p<0.05) compared to before the test. A comparison with control areas using placebo samples also showed a statistically significant difference (p<0.05), revealing that the ingredient **"Leuconostoc extracellular vesicles"** only contained in the test sample is effective for alopecia prevention. In addition, no special adverse reactions were observed in all study subjects, so it is considered a safe sample. (There may be temporary changes and individual differences.)

However, since this test is a simple test, it is difficult to say that the complete investigation of the sample is effective for alopecia preventionhas been made only by this test, and additional tests are considered necessary.

This test specifies that a total of 6 people, 1 person of responsible tester, 1 person of research institute director, and 4 persons of co-researchers, participated in the test directly from the start day of the test to the end day of the test.

KDRI Korea Dermatology Research Institute

The contents of this report are confidential and cannot be used for other purposes unless approved.
All or any part of the contents in this report cannot be copied or published without consent of KDRI.

10. References

a. KRAMER, Andreas, et al. How to prevent the detrimental effects of two months of bed-rest on muscle, bone and cardiovascular system: an RCT. *Scientific reports*, 2017, 7.1: 1-10.

b. HOLTHOFF, Vjera A., et al. Effects of physical activity training in patients with Alzheimer's dementia: results of a pilot RCT study. *PloS one*, 2015, 10.4: e0121478.

c. ANDERSSON, Ulf; CUERVO-CAZURRA, Alvaro; NIELSEN, Bo Bernhard. Explaining interaction effects within and across levels of analysis. In: Research methods in international business. Palgrave Macmillan, Cham, 2020. p. 331-349.

d. Baek et. al., Int. J. Cosmet. Sci., 2011, 33, 491-496

e. Choi JS, Park JB, Moon WS, Moon JN, Son SW, Kim MR., Safety and Efficacy of Rice Bran Supercritical CO2 Extract for Hair Growth in Androgenic Alopecia: A 16-Week Double-Blind Randomized Controlled Trial, Biol Pharm Bull. 2015;38(12):1856-63.

f. Jae-Hong Kim, Sung Yul Lee, Hae-Jin Lee, Na-Young Yoon and Won-Soo Lee, The Efficacy and Safety of 17α-Estradiol (Ell-Cranell® alpha 0.025%) Solution on Female Pattern Hair Loss: Single Center, Open-Label, Non-Comparative, Phase IV Study, Ann Dermatol. 2012 Aug; 24(3): 295–305.

g. Rachita Dhurat, Phototrichogram, Indian J Dermatol Venereol Leprol, May-June 2006, Vol 72, Issue 3, 242 -244

h. Won-Soo Lee, Hae-Jin Lee, Characteristics of Androgenetic Alopecia in Asian, Ann Dermatol Vol. 24, No. 3, 2012

i. Guideline for cosmetics applied to clinical test and efficacy test, Ministry of Food and Drug Safety of Korea, 2015.08

j. Guideline for substantiation of facts in labeling and advertising cosmetics. Ministry of Food and Drug Safety of Korea, 2018.03

k. Hyo-Jin Lee, M.D., Yang-Soo Kim, M.D., In Park, M.D., Calculation of Sample Size in Clinical Trials, Clinics in Shoulder and Elbow Volume 16, Number 1, June, 2013, 53-57

l. Guideline for cosmetic clinical study and efficacy test, Ministry of Food and Drug Safety of Korea, 2018.07

m. Hyun et. al., J. Soc. Cosmet. Scientists Korea, 2013, 39(3). 187-194

Korea Dermatology Research Institute

The contents of this report are confidential and cannot be used for other purposes unless approved.
All or any part of the contents in this report cannot be copied or published without consent of KDRI.

Attachment 1. Information of Test Institution

1-1. Test Personnel

A. Principal Investigator

Korea Dermatology Research Institute, Board Certified Dermatologist, Lee, Kyungreal, M.D.

B. Research Director

Korea Dermatology Research Institute, Lee, Donghwan

C. Research Members

Korea Dermatology Research Institute, Hong Jongwook.
Korea Dermatology Research Institute, Kim, Minji
Korea Dermatology Research Institute, Seo, Jihye
Korea Dermatology Research Institute, Kim, Jeongu

KDRI Korea Dermatology Research Institute

1-2. Reliability Assurance

A. Reliability Assurance Officer

Seoul National University Bundang Hospital, Department of Urology Assistant professor

Oh, Jongjin, M.D.

B. Persons in Charge of Reliability Assurance

Korea Dermatology Research Institute, Yoon, Miso
Korea Dermatology Research Institute, Kim, Jayoung

The contents of this report are confidential and cannot be used for other purposes unless approved.
All or any part of the contents in this report cannot be copied or published without consent of KDRI.

1-3. Research List of Test Institute

a. Evaluation and research on sun screen effect of cosmetic
b. Evaluation and research on skin wrinkles improvement effect of cosmetic
c. Evaluation and research on skin whitening effect of cosmetic
d. Evaluation and research on safety of cosmetic
e. Evaluation and research on other efficiencies of cosmetic
f. Evaluation and research on percutaneous absorption of effective substance
g. Sample analysis, effective substance extraction and research
h. Development and research on vehicle formulation
i. Development and research on other cosmetic related technologies

1-4. Facilities and Equipment of Test Institute

KDRI Korea Dermatology Research Institute

a. Air compressor
b. Anareobic chamber
c. ANTERA 3D
d. ARCO infrared thermometer AR-350 PLUS
e. Biologically weighted UV sensor with 8mm square adaptor for LLG with homogenizer
f. Biologically weighted UV sensor with LLG adaptor(SUV)
g. Blood Flow Imager OZ-2STD
h. Centrifuge
i. Chemi-doc
j. Chromameter CR-400®
k. Clean bench
l. Clinical photograph system
m. Constant Temperature and Humidity System
n. Constant Temperature and Humidity System HT-A5GG3
o. Corneometer® CM-825
p. Cutometer dual MPA 580
q. Cutometer® MPA-580
r. Cutometer® MPA-580 (2mm)
s. Cutometer® MPA-580 (8mm)

The contents of this report are confidential and cannot be used for other purposes unless approved.
All or any part of the contents in this report cannot be copied or published without consent of KDRI.

t. D-code system
u. DermaLab USB Moisture (Pin type)
v. DermaLab USB Ultrasound
w. Dermalite
x. Dermalite camera
y. Digital Camera system – DSLT, Macro Lens, Macro flash
z. Digital Hot Plates Wise Stirrer - MSH-20D
aa. Electronic balance – GF-4000, AF-220E
bb. ERYTHEMA UV & UVA INTENSITY METER MODEL 3D-600 V2.0
cc. F-RAY (Moire)
dd. FDC-6 Diffusion Cell Drive Console
ee. FLIR T-420
ff. Folliscope 2.8
gg. Folliscope 5.0
hh. Generall incubator 150L JSGI-150T
ii. Glossymeter® GL-200
jj. Incubator
kk. Infrared illuminator INFRALUX-300
ll. Ion Chef System
mm. Ion GeneStudio S5 plus
nn. IR Detector LP02 & LI19
oo. IR Detector PMA2100
pp. IR Detector PMA2140
qq. Janus Facial Image Analysis System
rr. JSAT-45 Autoclave
ss. Lambda 650S UV/Vis Spectrometer
tt. Mark-Vu
uu. Mexameter probe
vv. MicroCentrifuge
ww. MoistureMap MM100
xx. MoistureMeter D Compact
yy. MoistureMeter EpiD
zz. MPA 5
aaa. Multi Display Device MDD4

KDRI Korea Dermatology Research Institute

The contents of this report are confidential and cannot be used for other purposes unless approved.
All or any part of the contents in this report cannot be copied or published without consent of KDRI.

bbb. Multi Display Device MDD4

ccc. Multiport solar simulator 601 V2.5 300W

ddd. Multiport solar simulator 601-150W

eee. Olympus microscope, CX41-32C02

fff. PCR-C1000

ggg. PMA2100 Data Logging Meter Package

hhh. Polarized Dermoscopy – Dermlite-II pro camera kit

iii. Polarized Micro-scope c image analyzer

jjj. PRIMOS CR

kkk. Protein transfer

lll. Qubit™ 4 Fluorometer

mmm. SDS page electrophoresis

nnn. Sebufix® F 16 & Corneofix® F-20

ooo. Single-port solar simulator LS-1000

ppp. Skin-pH-meter® PH 905

qqq. Skin-Visiometer® SV-600

rrr. Skin-Visiometer® SV-700

sss. Tensile strength tester DS2-5N

ttt. Tensile strength tester system MR-PPS200

uuu. Tewameter TM Nano Probe

vvv. Tewameter® TM-300

www. Mexameter® MX-18

xxx. Sebumeter® SM-815

yyy. Translucency Meter TLS850

zzz. Ultrascan UC-22® cutis

aaaa. UVA sensor with 8mm square adaptor for LLG with homogenizer

bbbb. UVA sensor with LLG adaptor

cccc. Vapometer®

dddd. Vectra H2

eeee. VISIA-CR

ffff. Visioline VL650

gggg. Visioscan® VC 98

hhhh. hhhh. Waters alliance e2695 (HPLC)

Korea Dermatology Research Institute

The contents of this report are confidential and cannot be used for other purposes unless approved.
All or any part of the contents in this report cannot be copied or published without consent of KDRI.

Appendix 2. Information of Institutional Review Board (IRB)

A. Review Number : KDRI-IRB-20880

B. Review Result

Division	Review Date	Result	Note
**Plan Review**	2021. 03. 24	Approved	-
**Results Review**	2021. 07. 28	Approved	-

KDRI Korea Dermatology Research Institute

The contents of this report are confidential and cannot be used for other purposes unless approved.
All or any part of the contents in this report cannot be copied or published without consent of KDRI.

Appendix 3. Information Provided to the Subjects

Contact of the sub-investigator: Korea dermatology research institute / TEL.+82-31-704-0099

A. Purpose of test

This clinical trial is to evaluate the alopecia improvement effect of cosmetics and their safety in use.

B. Information on the test product

The test product is a cleaning product and is expected to have an acne alleviate effect. The control product consists only of excluding active ingredients. Test products and control products will be randomly assigned to the subjects participating in the test, and the assignment information will not be disclosed to the subjects until the end of the test. The probability that the study subjects belong to the control group by random assignment is 50%.

C. Precautions for research subjects

1) Follow the set number of uses and avoid excessive use.
2) Avoid excessive drinking and smoking during the test period.
3) Avoid excessive UV exposure during the test period.
4) Do not use cosmetics of the same type that have alopecia improvement effect during the test period.
5) If an abnormal reaction occurs during use, please contact the research center immediately.
6) You must keep the information obtained through this testing confidential until the end of the test period.
7) You must faithfully and accurately respond to any written inquiries, including questionnaires, provided during the test period.

D. Examination and procedure

Even after being selected as a research subject, if you are found unsuitable for the test during various pre-tests required for the test and a survey, you will not be able to participate in the test. If you are determined to be a suitable research subject, you will need to use the product twice a week for 12 weeks in the evening and will receive visual and device evaluations by experts at the center.

- Evaluation dates : Dates of visit ±5days
- Frequency : 5 times (Week 2 before start of the test, On week 0, 4, 8, 12)
- Test procedure (see the next page)

The contents of this report are confidential and cannot be used for other purposes unless approved.
All or any part of the contents in this report cannot be copied or published without consent of KDRI.

First Visit Day - Week 2 Before

- The subjects of the study
- Fill in the preliminary questionnaire and consent form after receiving the guidance on the test from the examiner
- A one scalp micropigmentation is performed on scalps with hair loss.
- Receive instructions on precautions and product usage, and receive products.

Second Visit Day - Week 2

- Phototrichogram focusing on scalp micropigmentation and photography of the scalp and hair
- Offer the sample.

Third Visit Day – Week 4

- Phototrichogram focusing on scalp micropigmentation and photography of the scalp and hair
- Visual evaluation by experts and self-assessment by subjects
- Evaluate any side effects of the product by dermatologists

KDRI Korea Dermatology Research Institute

Fourth Visit Day – Week 6

- Phototrichogram focusing on scalp micropigmentation and photography of the scalp and hair
- Visual evaluation by experts and self-assessment by subjects
- Evaluate any side effects of the product by dermatologists

Fifth Visit Day – Week 8

- Phototrichogram focusing on scalp micropigmentation and photography of the scalp and hair
- Visual evaluation by experts and self-assessment by subjects
- Evaluate any side effects of the product by dermatologists
- After the test is over, you will receive a test participation fee

E. Risks or discomfort that may be expected in the research study subjects

Skin irritation such as erythema, edema, scaling, stinging, burning, tightness, prickling, and rash as well as unknown side effects may occur during the test. When new information on safety is collected during research, it will be provided to the subjects or their representatives in a timely manner.

The contents of this report are confidential and cannot be used for other purposes unless approved.
All or any part of the contents in this report cannot be copied or published without consent of KDRI.

F. Appropriate contraceptive methods for women

For this test, pregnant women, lactating women, or pregnant women who have a plan to conceive or disagree with the appropriate choice of contraception suggested below are not permitted to participate in the test.

- Intrauterine contraceptive devices: loops, etc.
- Blocking contraception: femidome, vaginal pesticides, etc.

G. Compensation and benefits for using the product

The product used for all exams and tests scheduled for this test plan are provided free of charge. If you successfully complete the test, you will be paid for your participation. (However, if you are dropped from the test, the amount paid may be adjusted according to the guidelines of the research center.)

H. Treatment for research-related injuries in subjects

In the event that research-related injuries occur to subjects even if they have faithfully followed the instructions, the sponsor will take legal responsibility and provide compensation for the injuries according to the relevant regulations, and the subjects will receive the best treatment at medical institutions affiliated with the research center and other medical institutions.

KDRI Korea Dermatology Research Institute

I. Voluntary participation and voluntary dropout

Study subjects can freely decide whether to participate in the research, and no penalty will be given to an individual's free decision to drop out.

J. Matters related to privacy and data access

Photos and related data obtained from study subjects through this test can be used for purposes such as cosmetic and medical research and advertising, but all records that can reveal a participant's identity will be kept confidential even when the test results are published in the future.

Reliability assurance personnel and the Minister of Food and Drug Safety may access the records of the study subjects in order to verify the reliability of the test procedures and data within the scope prescribed by relevant regulations without violating the confidentiality of study subjects. Upon signing the agreement, subjects will be deemed to consent to the above.

K. Notifications for study subjects

If new facts about the test product, which may affect a study subject's decision to participate in the testing, are discovered during the test period, the information will be announced without delay.

The contents of this report are confidential and cannot be used for other purposes unless approved.
All or any part of the contents in this report cannot be copied or published without consent of KDRI.

L. Study subjects may be dropped from the test in the following cases:

1) When a subject shows excessive adverse reactions such as itching or erythema on the test areas
2) When the representative of a subject such as a guardian requests discontinuation of the test
3) When a subject or the investigator seriously violates the test plans and precautions
4) Other cases the investigator considers inappropriate for continuance of the test

M. Number of study subjects

More than 10 (including dropouts)

N. Additional information regarding the test, the rights and interests of study subjects, and the personnel to contact to report injuries

Minji Kim (Contact: +82-31-704-0099)

KDRI Korea Dermatology Research Institute

The contents of this report are confidential and cannot be used for other purposes unless approved.
All or any part of the contents in this report cannot be copied or published without consent of KDRI.

Appendix 4. Compensation for Research Related Injuries

Contact information of Compensation for research related injuries : Korea dermatology research institute / TEL. +82-31-704-0099

A. Basic principles

1) In the event of "injuries to subjects caused by participation in human subject research," the research center and the sponsor shall ensure that appropriate and prompt compensation is provided according to their standards, procedures, and related laws for compensation.
2) If injuries occur to study subjects during the test period, the principal investigator (director) shall first help them receive treatment from a designated medical institution, prescribe ointment, and explain about the compensation process, and then identify the cause of the injuries.
3) The subjects submit an "application for injury compensation," and the principal investigator (director) fills out a "compensation record" and provides appropriate compensation according to the cause of the damage.
4) Damage compensation for the subjects does not apply to cases where injuries were caused intentionally or by negligence, and such "compensation" is subject to matters set by other laws including the Civil Act. "Compensation" does not necessarily cover all medical expenses but is generally made based on standards predetermined by the compensators (the institution conducting the human subject research and the sponsor).
5) The principal investigator (director) shall provide the subjects with detailed information about the compensation or treatment method to be given in the event of research-related injuries when obtaining their voluntary consent before the test in accordance with Item 7, No. 10 (J) of the Korean Good Clinical Practice of the「Regulation on Safety of Medicinal Products」and offer information on compensation upon the request of subjects.
6) In the event of research-related physical injuries to subjects, the principal investigator (director) shall first provide appropriate treatment or treatment opportunities even before financial compensation is determined.

B. Definition of injury caused by participation in human subject research

"Injury caused by participation in human subject research" refers to injuries caused by samples used for human subject research conducted in accordance with the clinical trial protocol or by physicochemical or medical intervention performed in accordance with the protocol, which would not have occurred if it had not been for the human subject research.
*Examples of injuries: erythema, edema, scaling, itching, stinging, burning, tightness, prickling, and other abnormal symptoms

C. Those who are subject to the compensation policies for human subject research

The contents of this report are confidential and cannot be used for other purposes unless approved.
All or any part of the contents in this report cannot be copied or published without consent of KDRI.

Those who are subject to the compensation policies and not excluded from compensation are as follows:

1) Subjects who had physical injuries (defined in Section 4) due to participation in human subject research
2) Subjects who had physical injuries that would not have occurred if it had not been for human subject research
3) Subjects who had injuries during treatment of adverse reactions caused by participation in human subject research
4) Even if the injury had been expected and subjects voluntarily agreed to participate in the human subject research, they are eligible for compensation

D. Cases exempt from the compensation policies for human subject research

1) Those whose physical injuries are not causally connected to the human subject research
2) Those with physical injuries that may have occurred due to other causes
3) When the expected effect has not appeared by the sample for the human subject research or other benefits have not been provided
4) When it is not possible to provide the effect of the human subject research to those who received the placebo sample
5) If study subjects or their guardians have not complied with the investigator's instructions and the research protocol intentionally or through gross negligence, the amount of compensation may be reduced or they can be excluded from compensation

KDRI Korea Dermatology Research Institute

E. Compensation procedure for research-related injuries

The principal investigator (director) must provide a written explanation of the compensation application process in the consent and instruction form and explain the process before obtaining consent from study subjects to participate in human subject research so that they can receive proper compensation for any possible injuries incurred during the test.

1) Upon a request for compensation from subjects, the principal investigator (director) must record the details and notify the sponsor if the case is related to side effects caused by the test sample.
2) The principal investigator (director) investigates whether compensation is applicable and determines the level of compensation according to the evaluation criteria.
3) The principal investigator (director) must inform the study subject of the result of compensation as soon as possible.
4) If the study subject does not agree to the level of compensation, a third party (dermatologists) will re-evaluate the case.

After compensation has been made, all information related to the compensation is recorded and signed. - Cases of side effects, treatment and compensation for each side effect

The contents of this report are confidential and cannot be used for other purposes unless approved.
All or any part of the contents in this report cannot be copied or published without consent of KDRI.

Attachment 5. Appendix Image Data

1) Clinical photos –Test group

ID		Week 0	Week 4	Week 8	Week 12
2603	Vertex	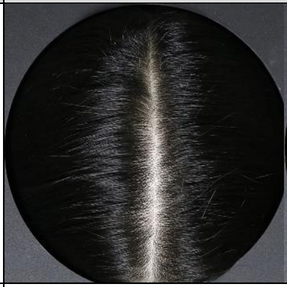	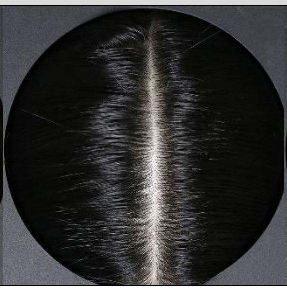	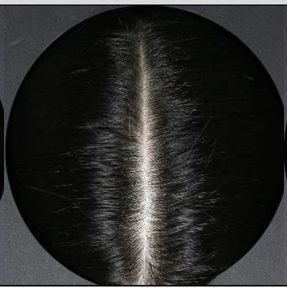	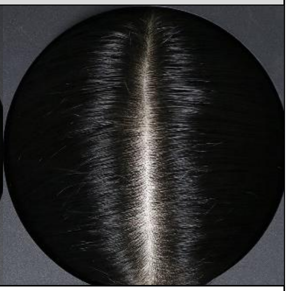
	Hairline	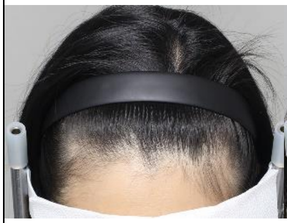	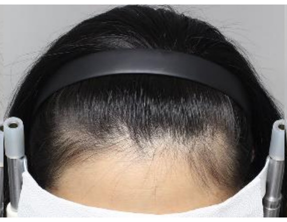	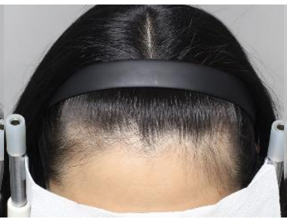	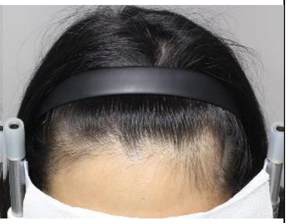
2793	Vertex	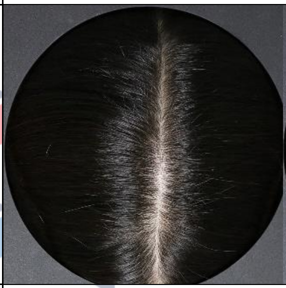	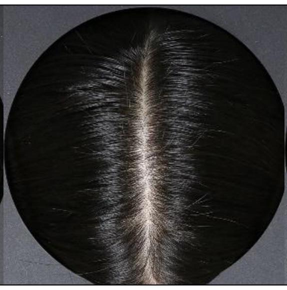	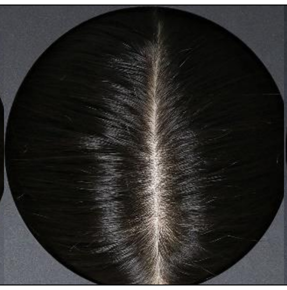	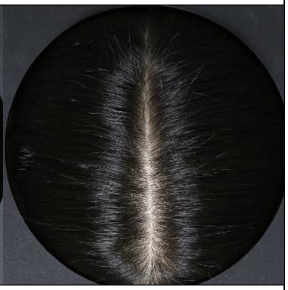
	Hairline	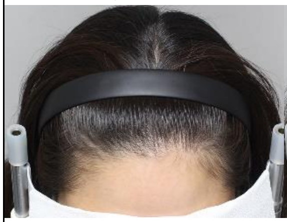	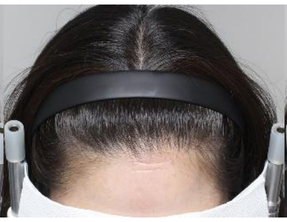	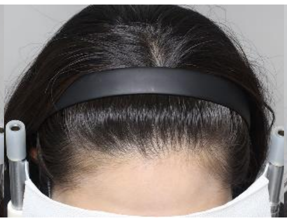	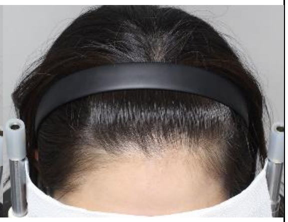
1984	Vertex	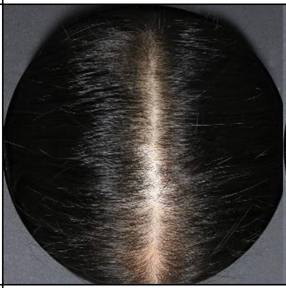	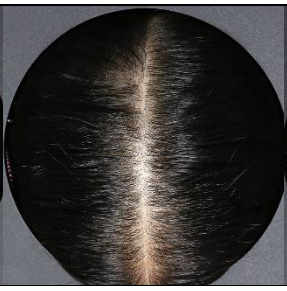	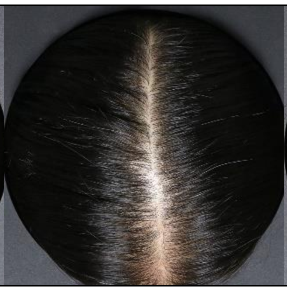	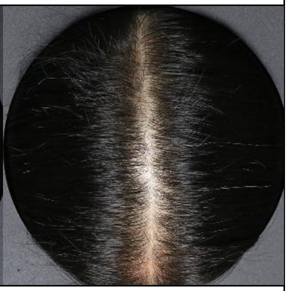
	Hairline	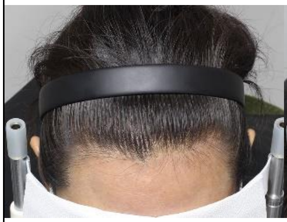	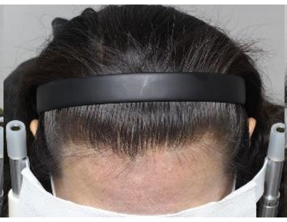	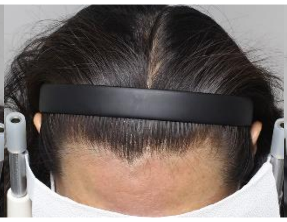	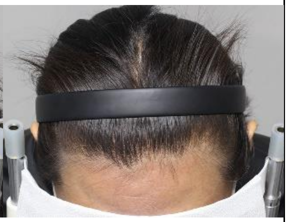

The contents of this report are confidential and cannot be used for other purposes unless approved.
All or any part of the contents in this report cannot be copied or published without consent of KDRI.

ID		Week 0	Week 4	Week 8	Week 12
3940	Vertex	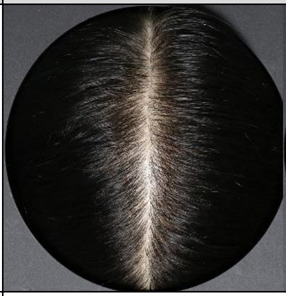	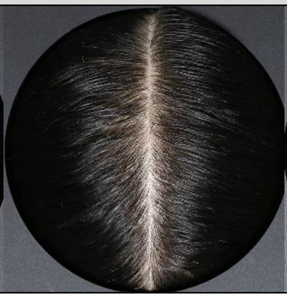	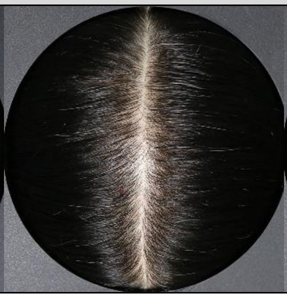	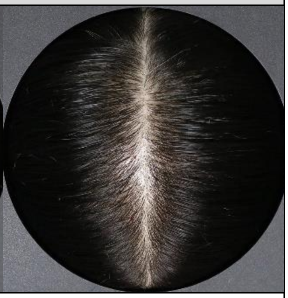
	Hairline	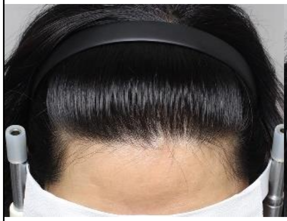	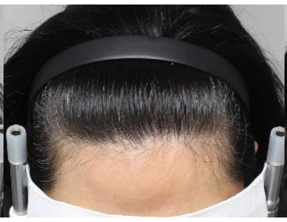	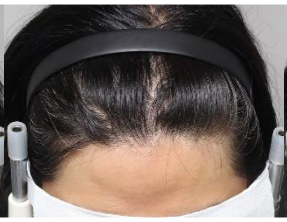	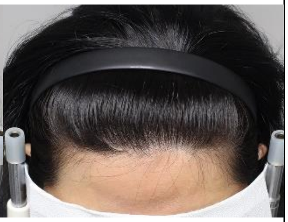
3238	Vertex	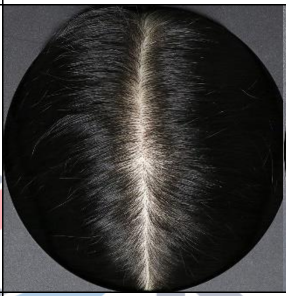	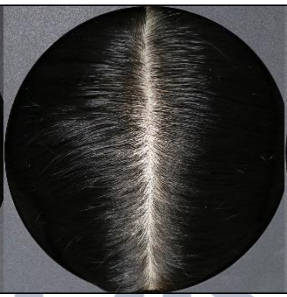	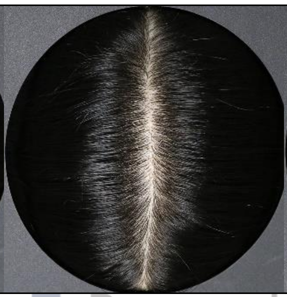	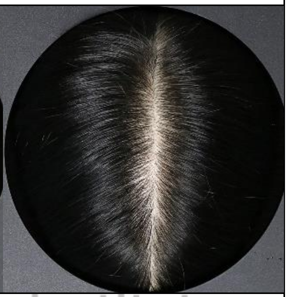
	Hairline	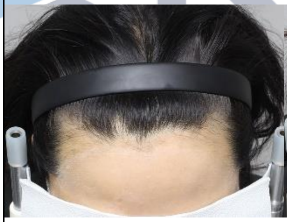	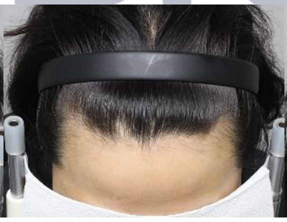	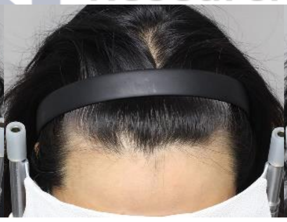	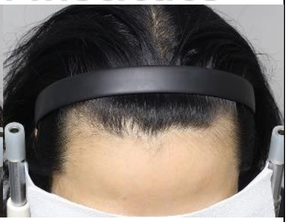
3822	Vertex	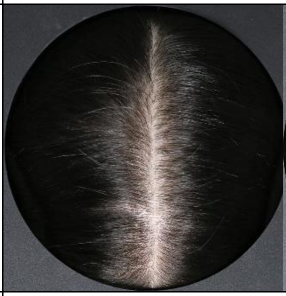	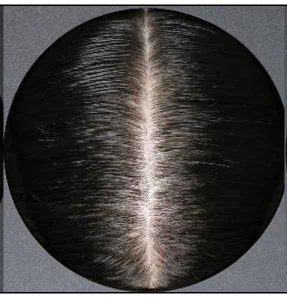	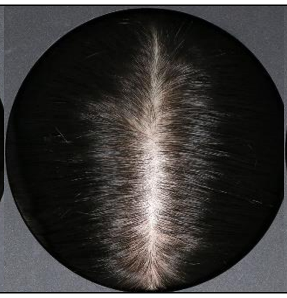	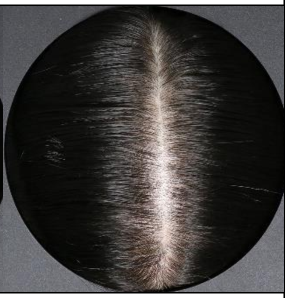
	Hairline	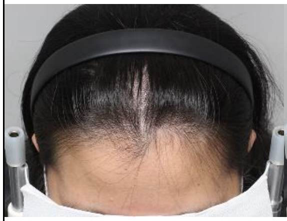	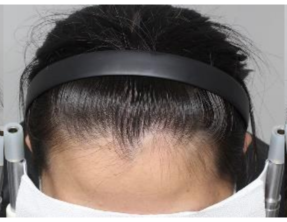	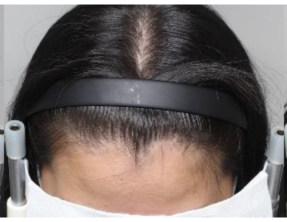	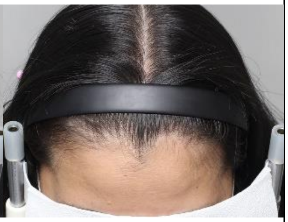

Research Institute

The contents of this report are confidential and cannot be used for other purposes unless approved.
All or any part of the contents in this report cannot be copied or published without consent of KDRI.

ID		Week 0	Week 4	Week 8	Week 12
3943	Vertex	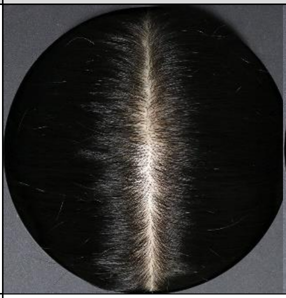	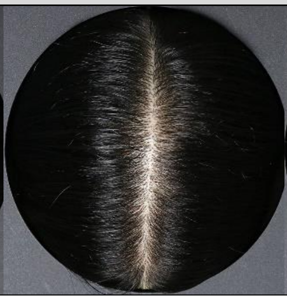	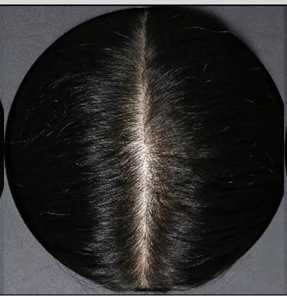	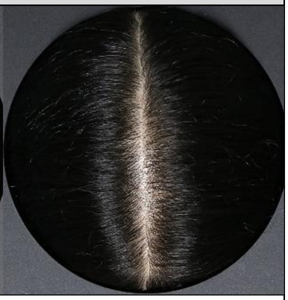
	Hairline	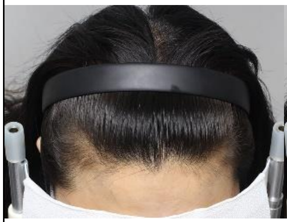	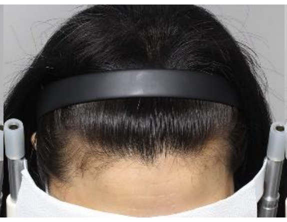	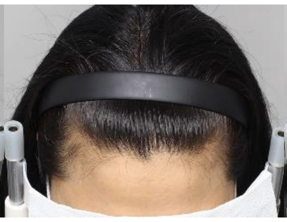	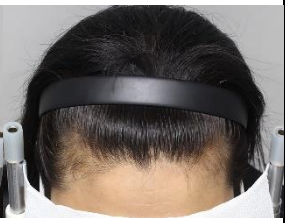
4167	Vertex	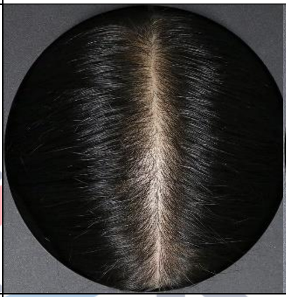	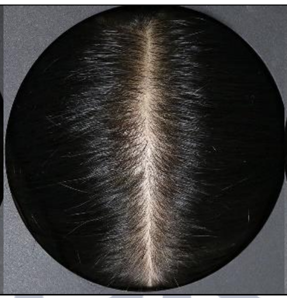	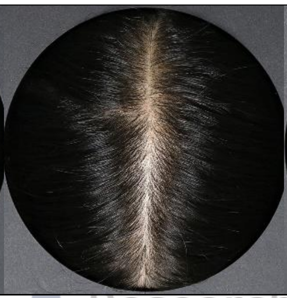	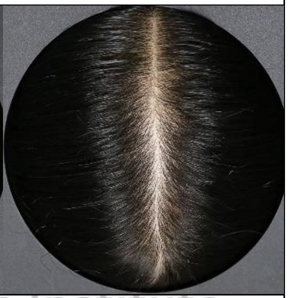
	Hairline	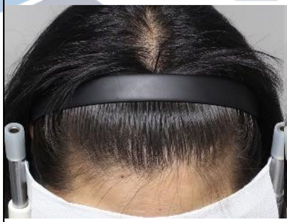	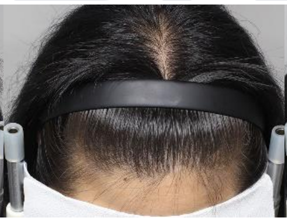	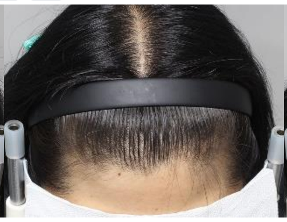	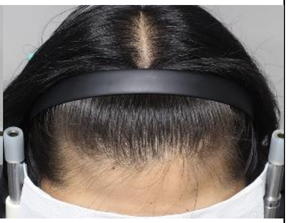
4203	Vertex	-	-	-	-
	Hairline	-	-	-	-

The contents of this report are confidential and cannot be used for other purposes unless approved.
All or any part of the contents in this report cannot be copied or published without consent of KDRI.

ID		Week 0	Week 4	Week 8	Week 12
3668	Vertex	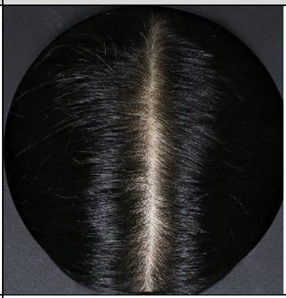	-	-	-
	Hairline	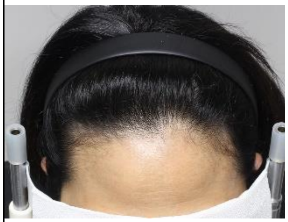			
4005	Vertex	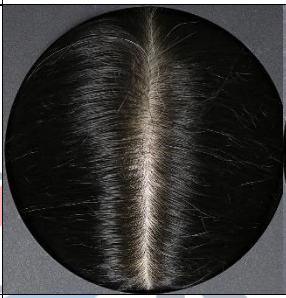	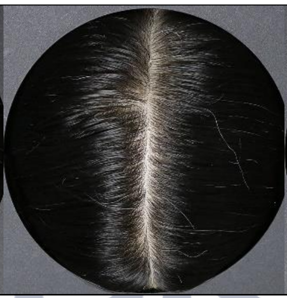	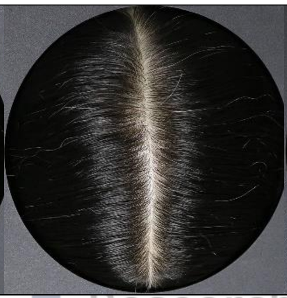	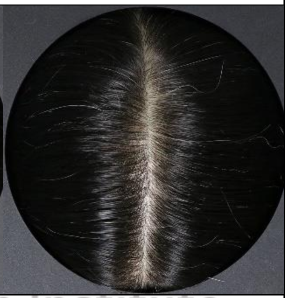
	Hairline	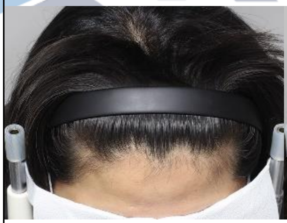	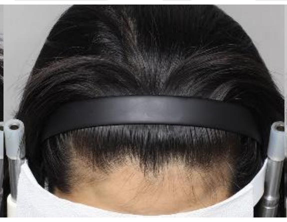	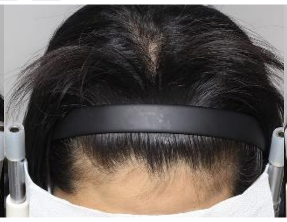	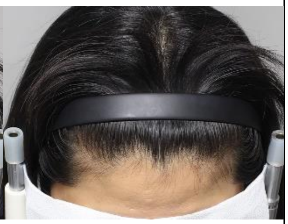
1719	Vertex	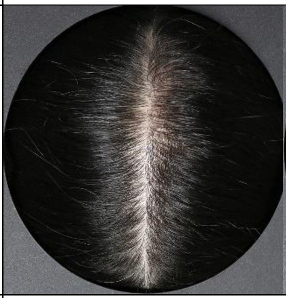	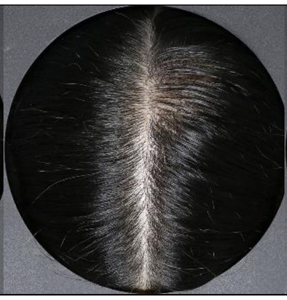	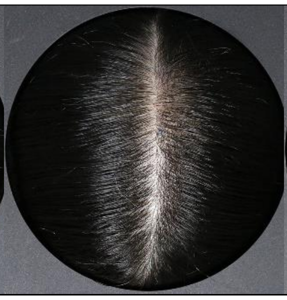	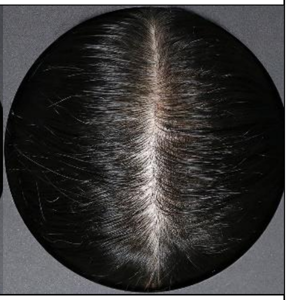
	Hairline	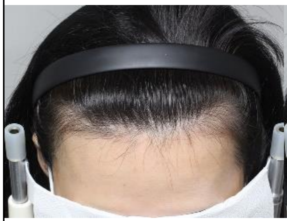	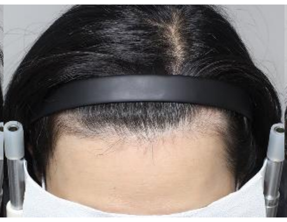	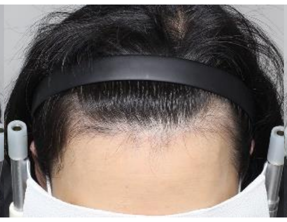	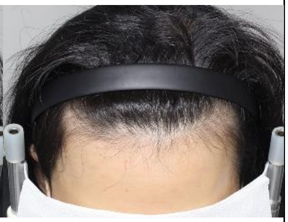

KDRI Research Institute

The contents of this report are confidential and cannot be used for other purposes unless approved.
All or any part of the contents in this report cannot be copied or published without consent of KDRI.

ID		Week 0	Week 4	Week 8	Week 12
897	Vertex	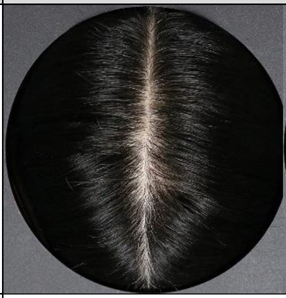	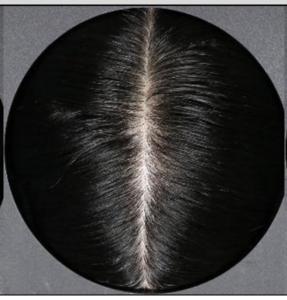	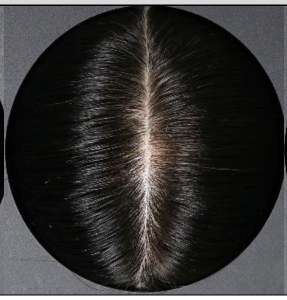	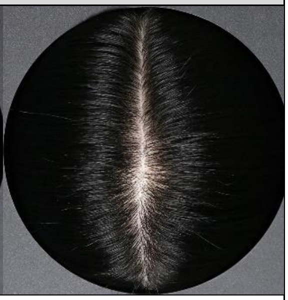
	Hairline	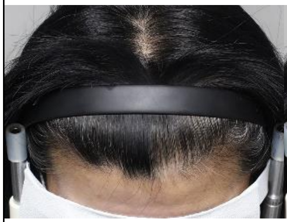	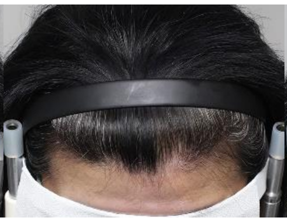	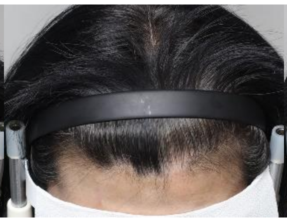	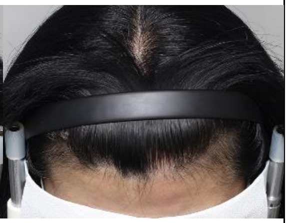
3964	Vertex	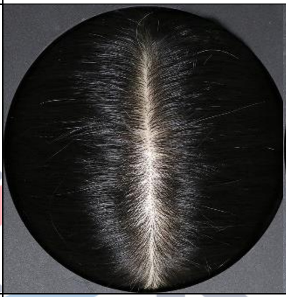	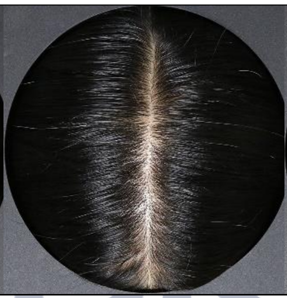	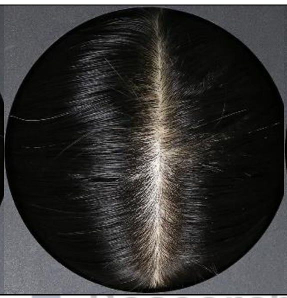	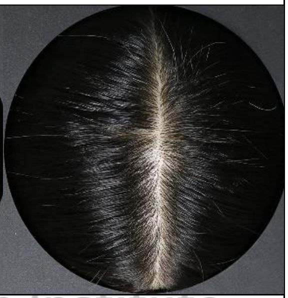
	Hairline	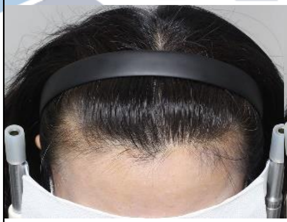	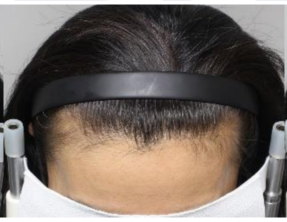	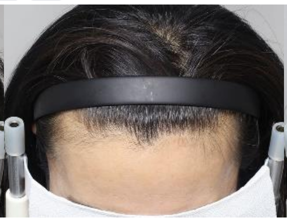	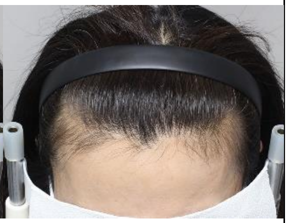
1297	Vertex	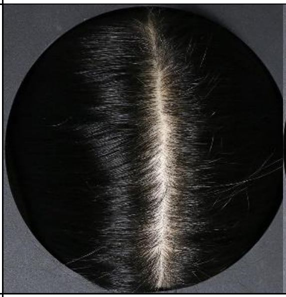	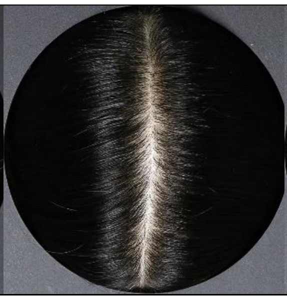	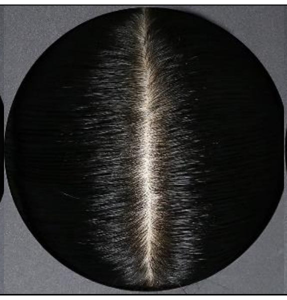	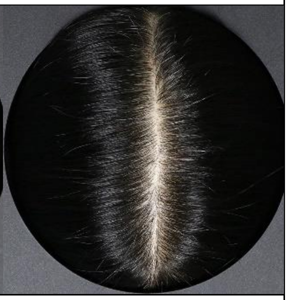
	Hairline	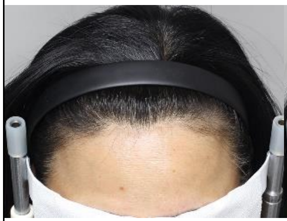	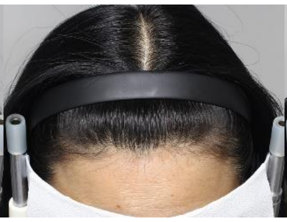	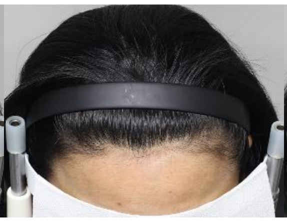	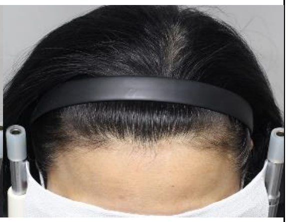

KDRI Research Institute

The contents of this report are confidential and cannot be used for other purposes unless approved.
All or any part of the contents in this report cannot be copied or published without consent of KDRI.

ID		Week 0	Week 4	Week 8	Week 12
5646	Vertex	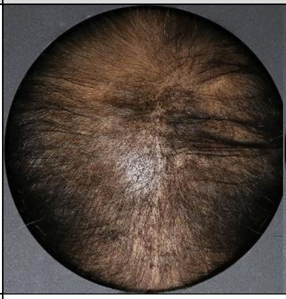	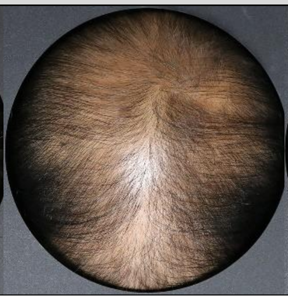	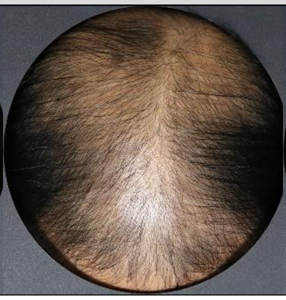	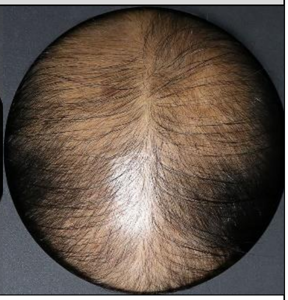
	Hairline	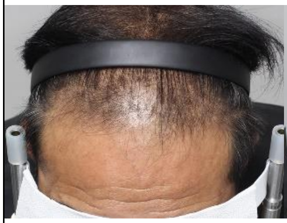	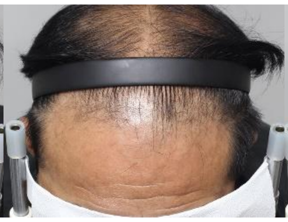	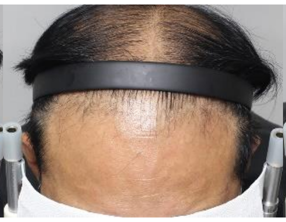	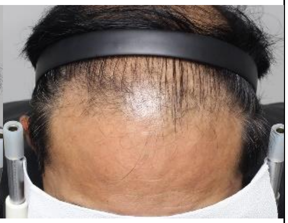
4997	Vertex	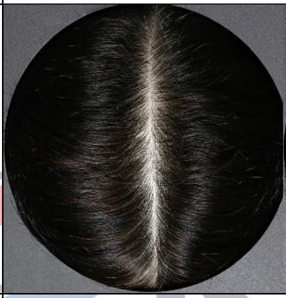	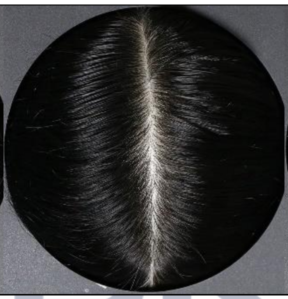	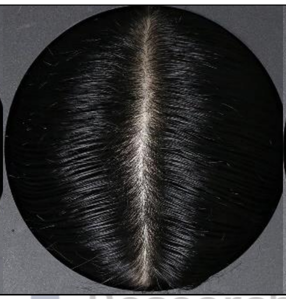	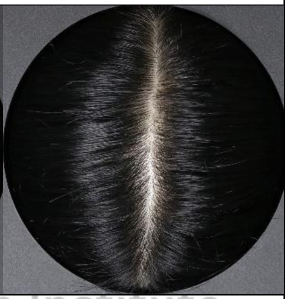
	Hairline	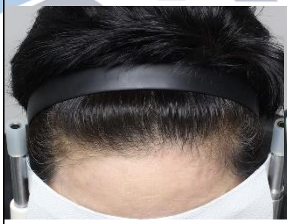	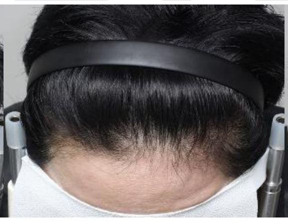	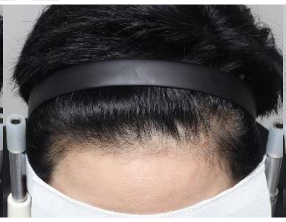	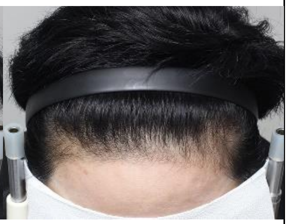

KDRI Research Institute

The contents of this report are confidential and cannot be used for other purposes unless approved.
All or any part of the contents in this report cannot be copied or published without consent of KDRI.

2) Clinical photos –Placebo group

ID		Week 0	Week 4	Week 8	Week 12
5182	Vertex	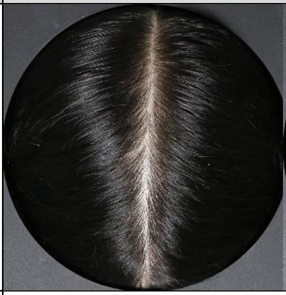	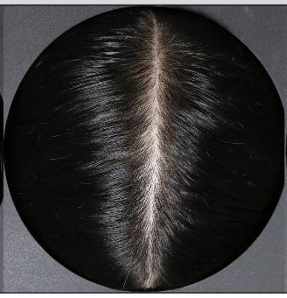	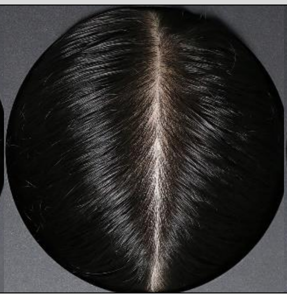	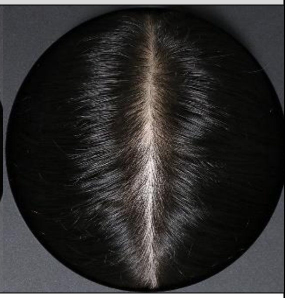
	Hairline	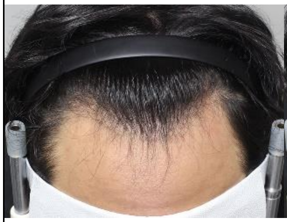	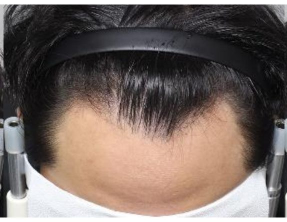	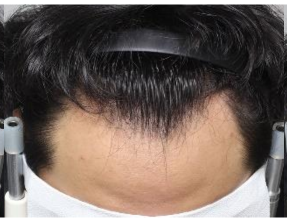	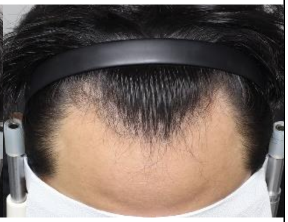
4567	Vertex	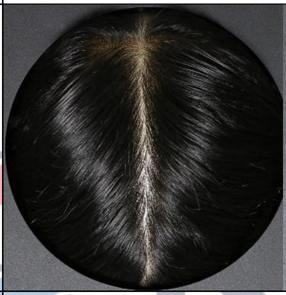	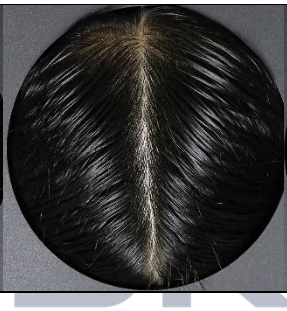	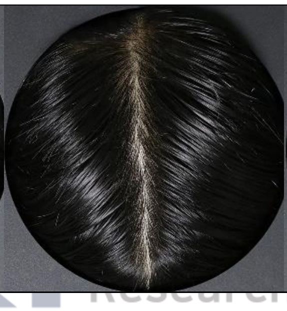	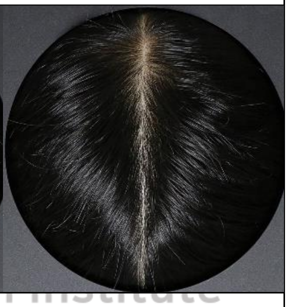
	Hairline	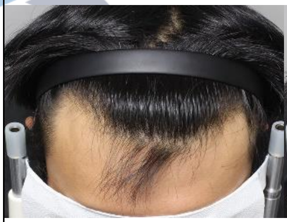	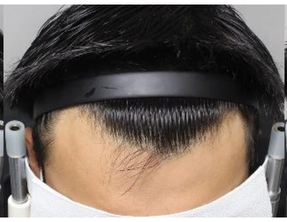	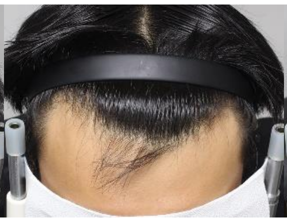	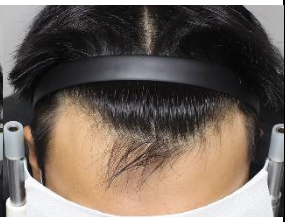
2884	Vertex	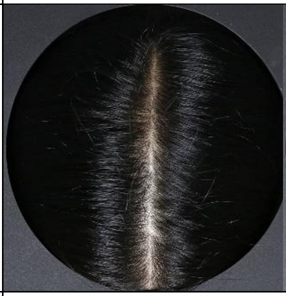	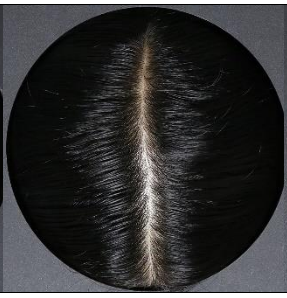	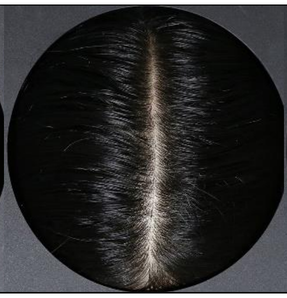	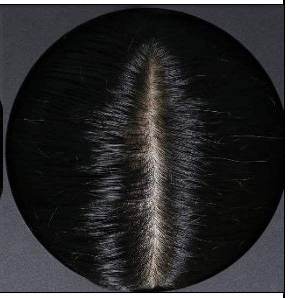
	Hairline	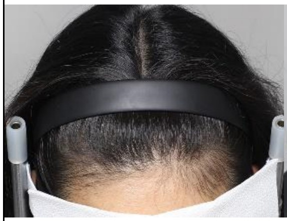	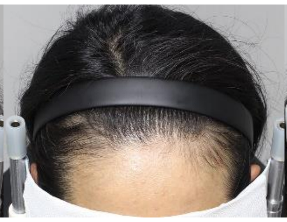	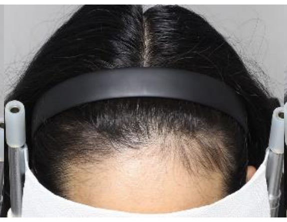	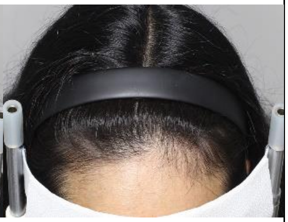

The contents of this report are confidential and cannot be used for other purposes unless approved.
All or any part of the contents in this report cannot be copied or published without consent of KDRI.

ID		Week 0	Week 4	Week 8	Week 12
4014	Vertex	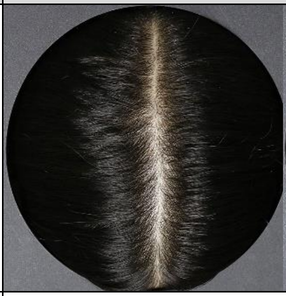	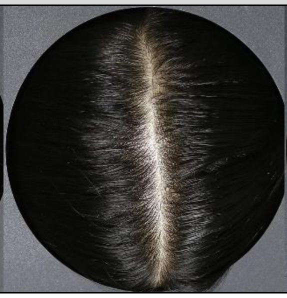	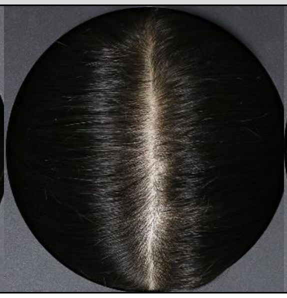	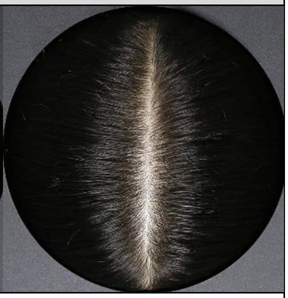
	Hairline	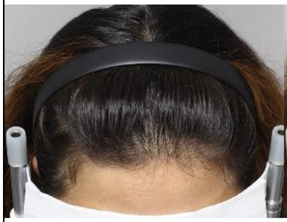	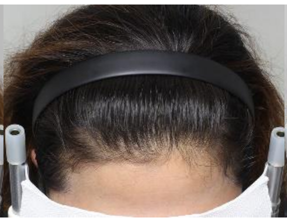	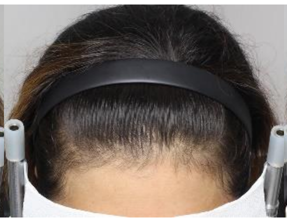	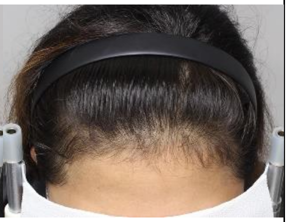
2498	Vertex	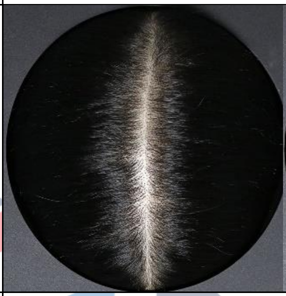	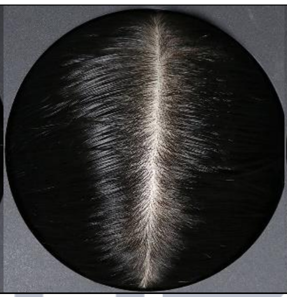	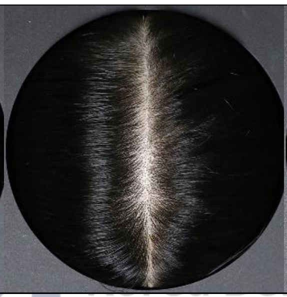	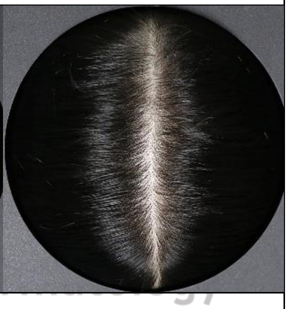
	Hairline	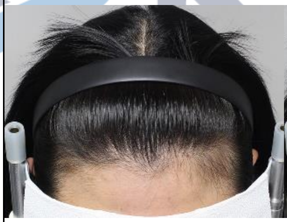	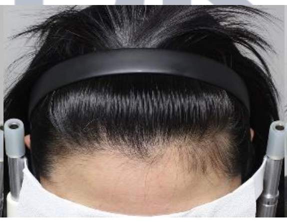	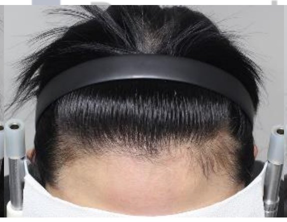	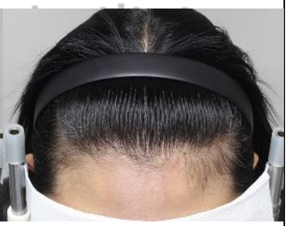
2379	Vertex	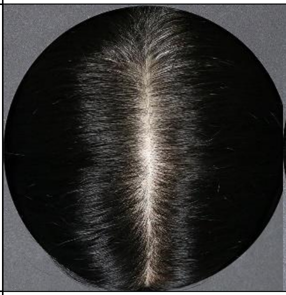	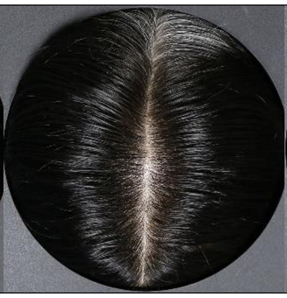	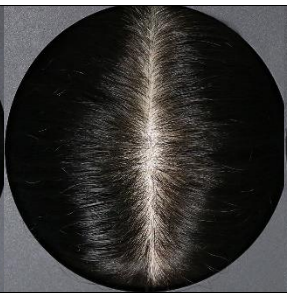	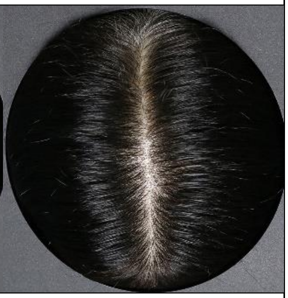
	Hairline	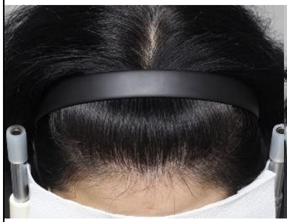	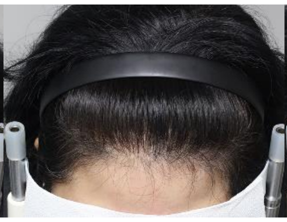	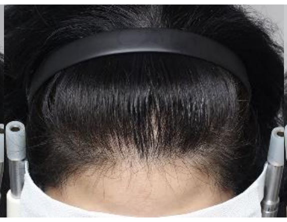	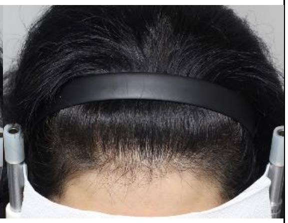

The contents of this report are confidential and cannot be used for other purposes unless approved.
All or any part of the contents in this report cannot be copied or published without consent of KDRI.

ID		Week 0	Week 4	Week 8	Week 12
763	Vertex	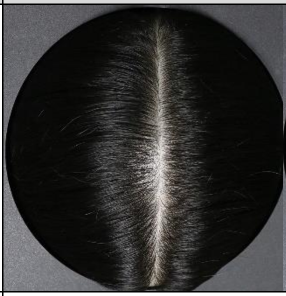	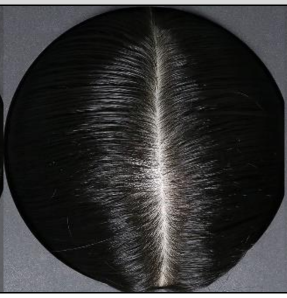	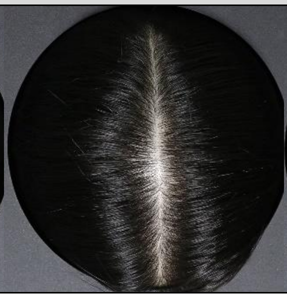	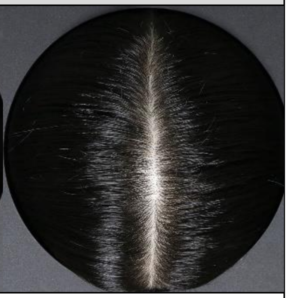
	Hairline	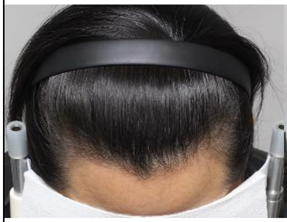	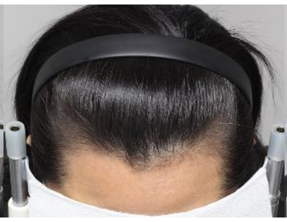	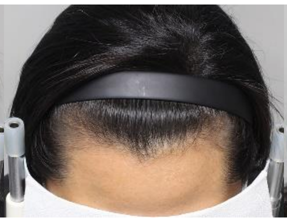	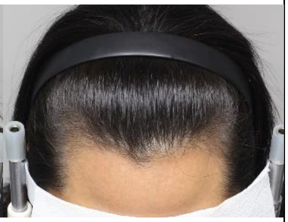
2515	Vertex	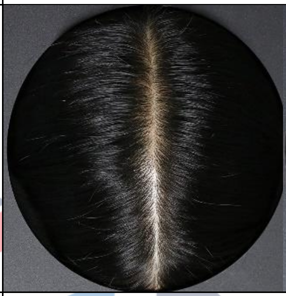	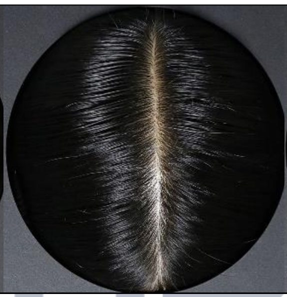	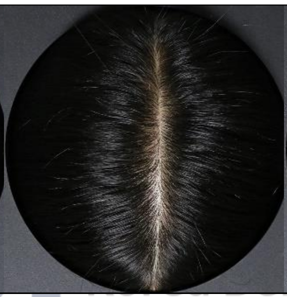	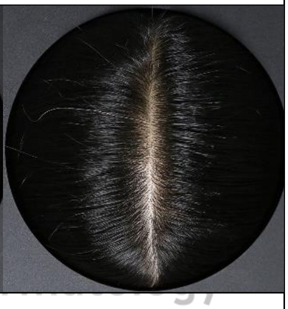
	Hairline	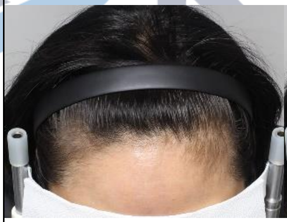	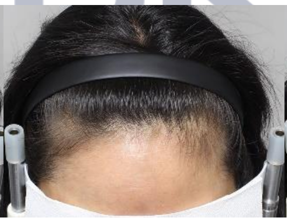	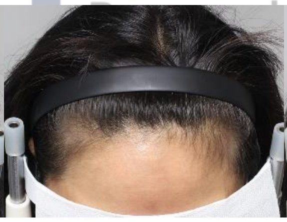	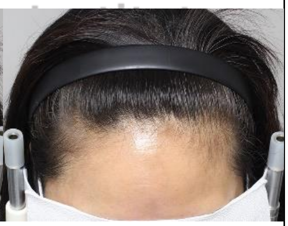
2984	Vertex	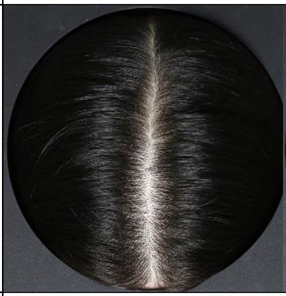	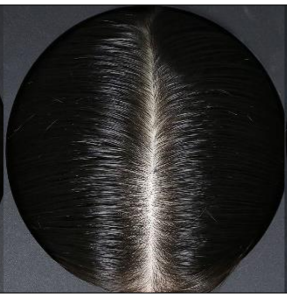	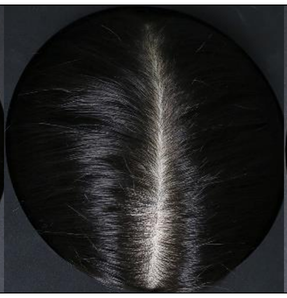	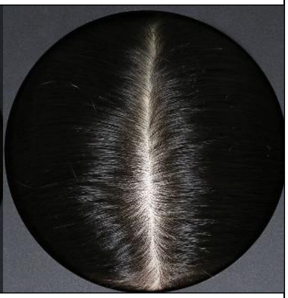
	Hairline	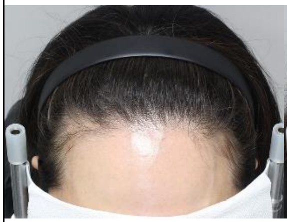	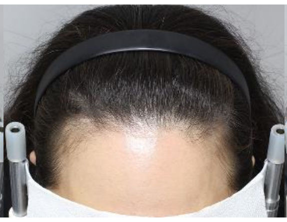	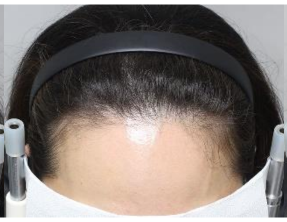	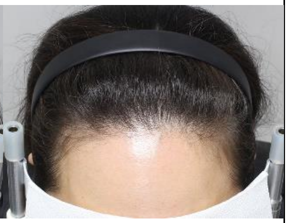

The contents of this report are confidential and cannot be used for other purposes unless approved.
All or any part of the contents in this report cannot be copied or published without consent of KDRI.

ID		Week 0	Week 4	Week 8	Week 12
5648	Vertex	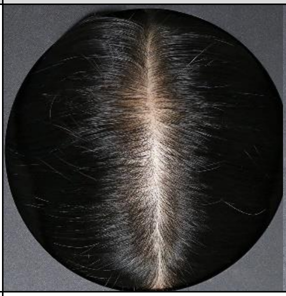	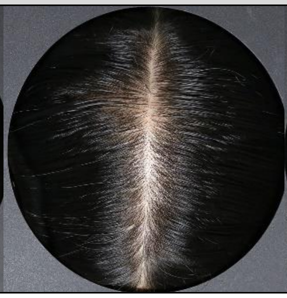	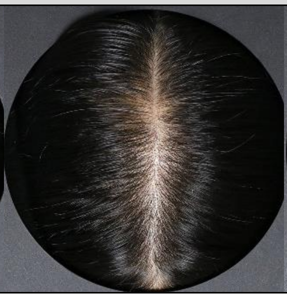	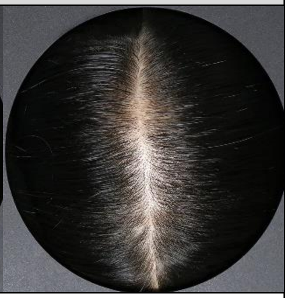
	Hairline	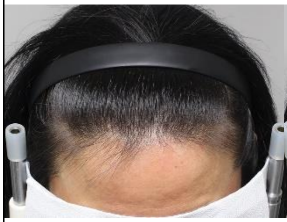	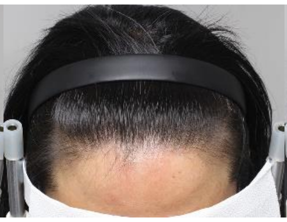	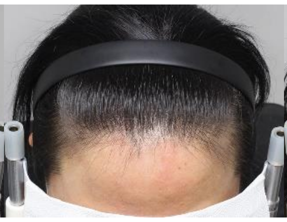	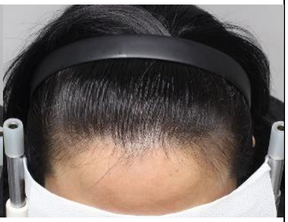
2909	Vertex	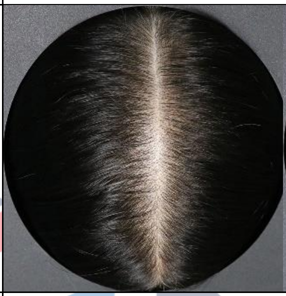	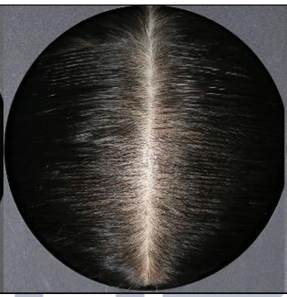	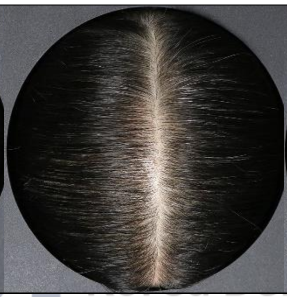	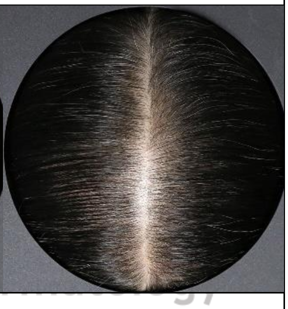
	Hairline	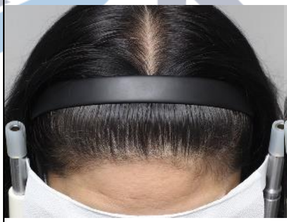	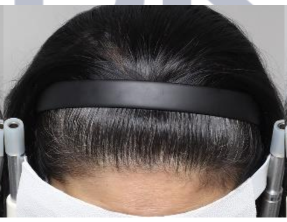	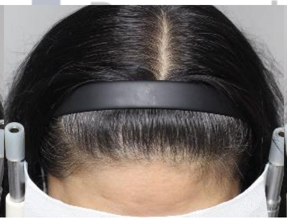	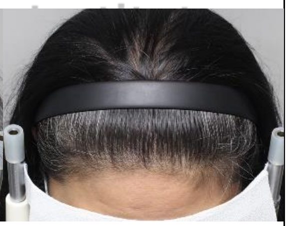
4419	Vertex	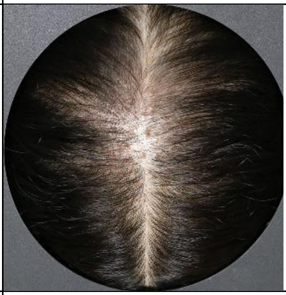	-	-	-
	Hairline	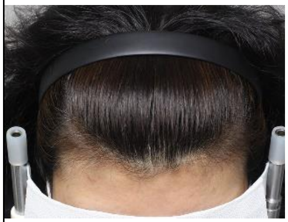	-	-	-

The contents of this report are confidential and cannot be used for other purposes unless approved.
All or any part of the contents in this report cannot be copied or published without consent of KDRI.

ID		Week 0	Week 4	Week 8	Week 12
1482	Vertex	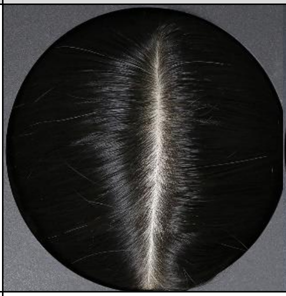	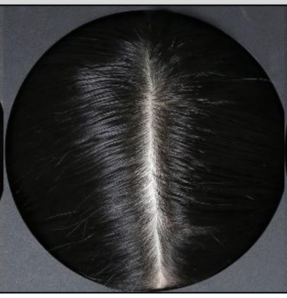	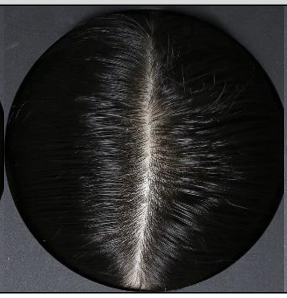	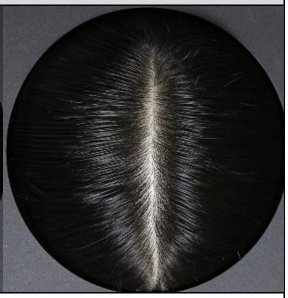
	Hairline	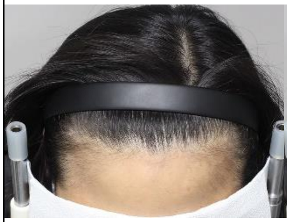	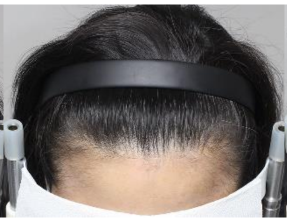	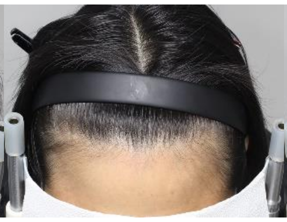	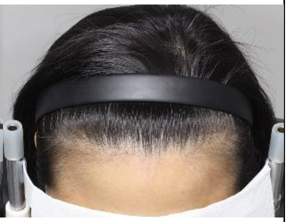
4303	Vertex	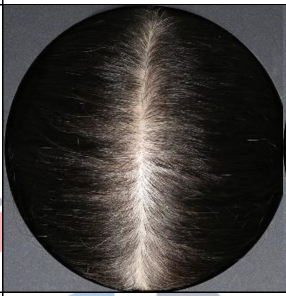	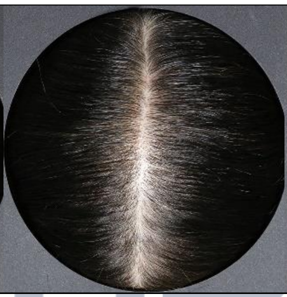	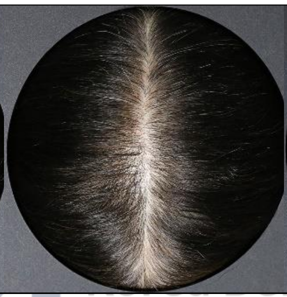	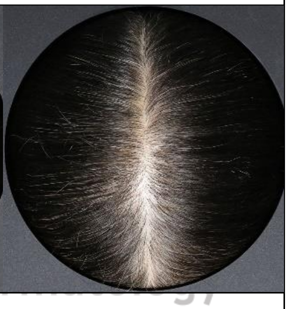
	Hairline	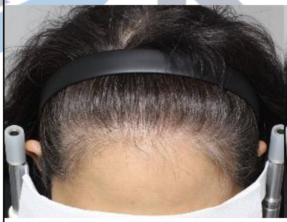	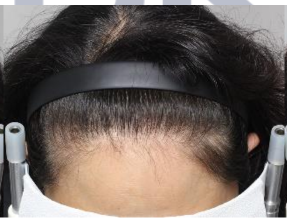	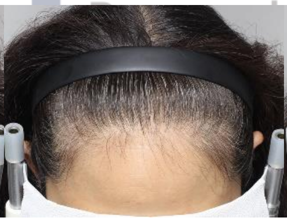	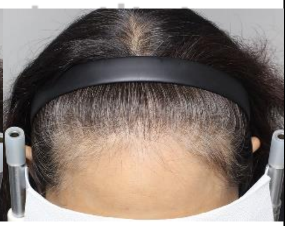
4318	Vertex	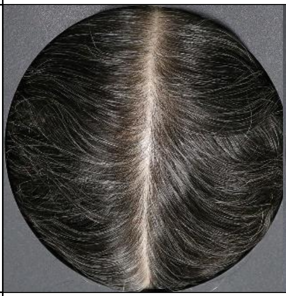	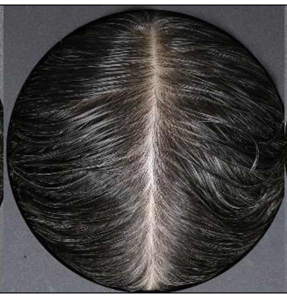	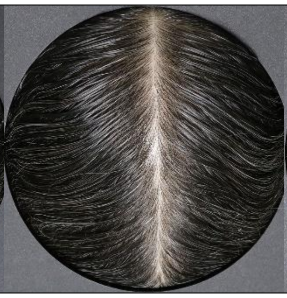	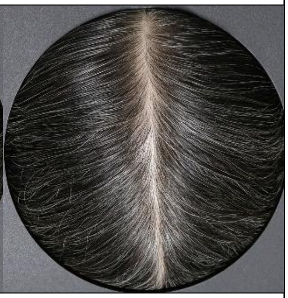
	Hairline	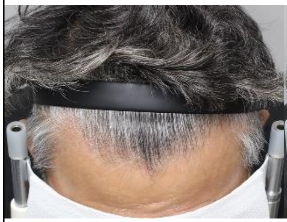	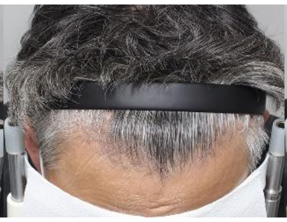	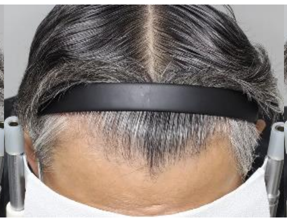	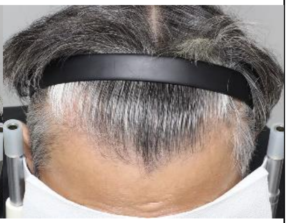

The contents of this report are confidential and cannot be used for other purposes unless approved.
All or any part of the contents in this report cannot be copied or published without consent of KDRI.

ID		Week 0	Week 4	Week 8	Week 12
4077	Vertex	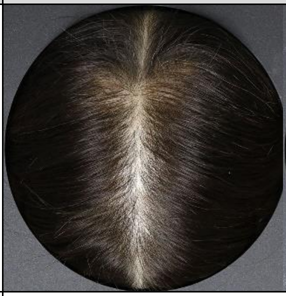	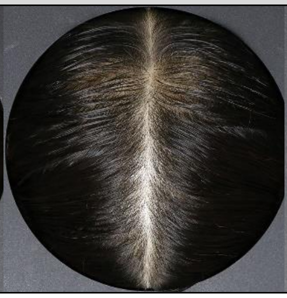	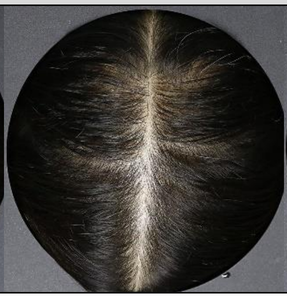	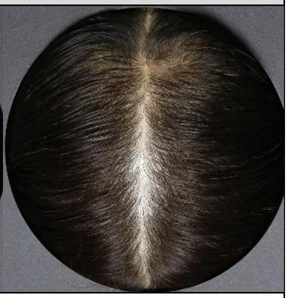
	Hairline	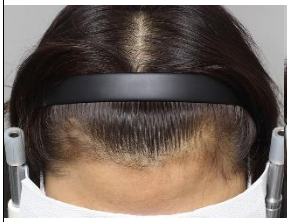	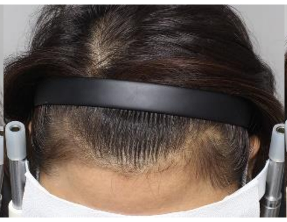	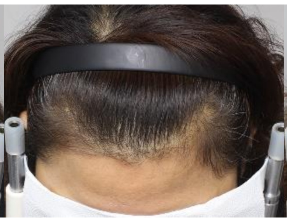	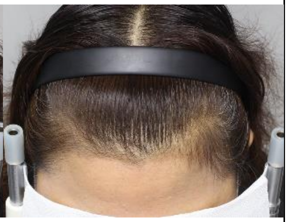
2824	Vertex	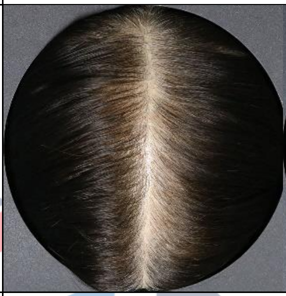	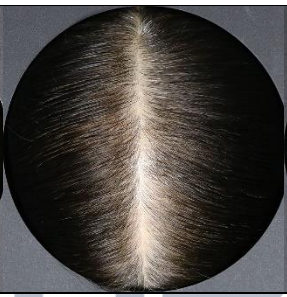	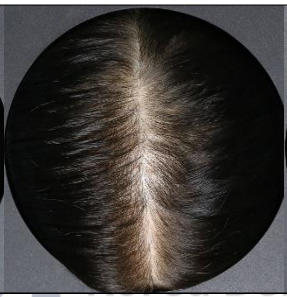	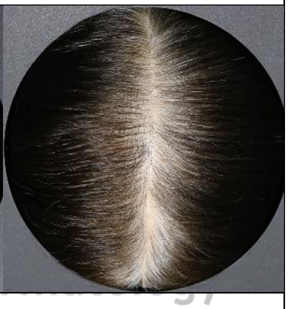
	Hairline	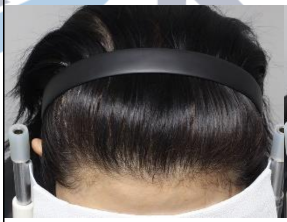	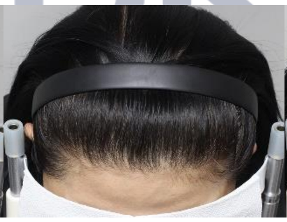	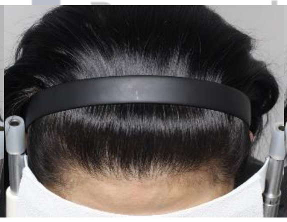	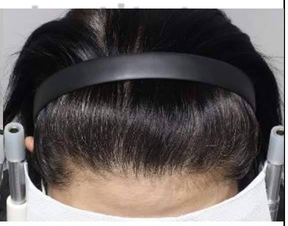

The contents of this report are confidential and cannot be used for other purposes unless approved.
All or any part of the contents in this report cannot be copied or published without consent of KDRI.

3) Phototrichogram Image –Test group

ID	Week 0	Week 4	Week 8	Week 12
2603	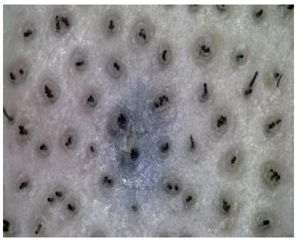	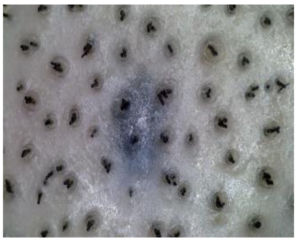	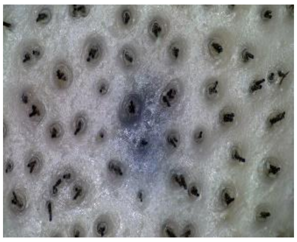	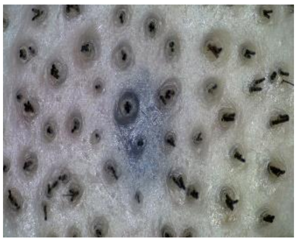
2793	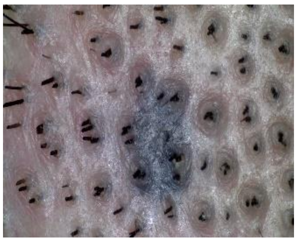	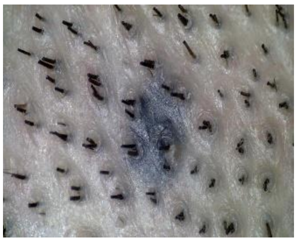	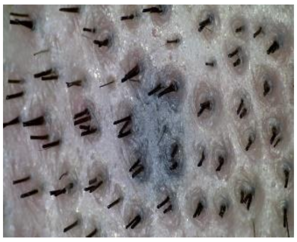	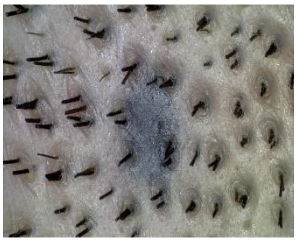
1984	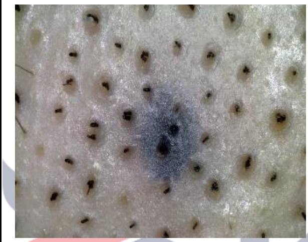	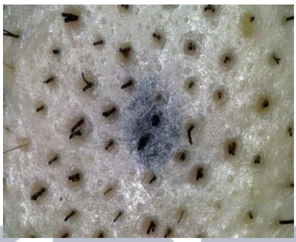	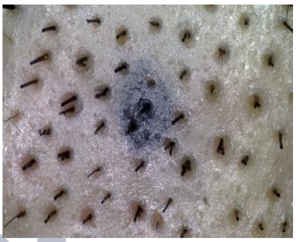	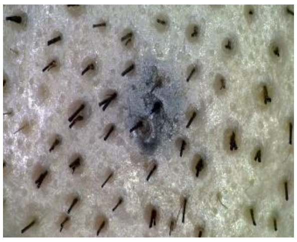
3940	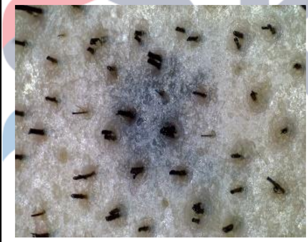	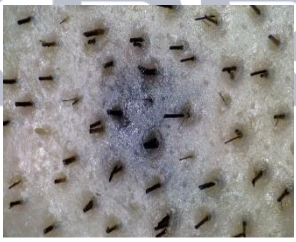	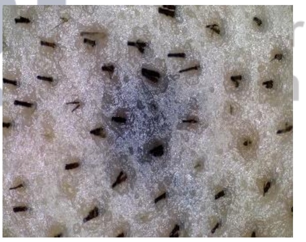	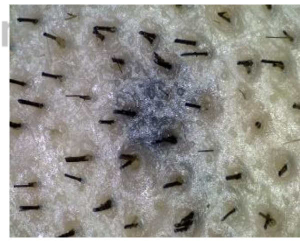
3238	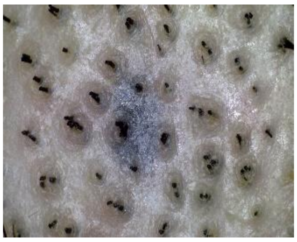	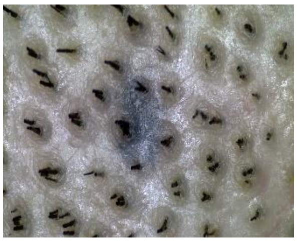	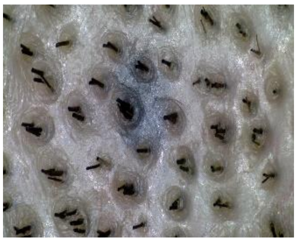	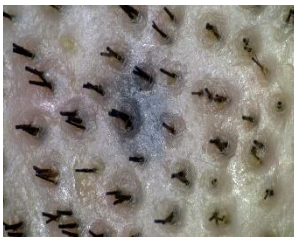
3822	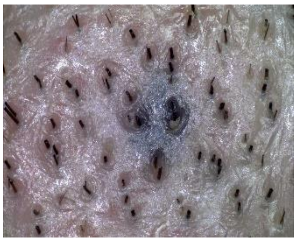	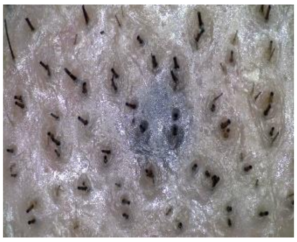	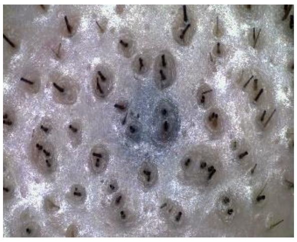	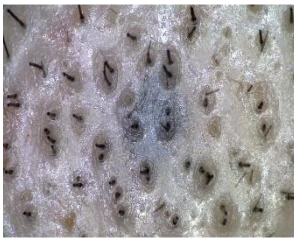

The contents of this report are confidential and cannot be used for other purposes unless approved.
All or any part of the contents in this report cannot be copied or published without consent of KDRI.

ID	Week 0	Week 4	Week 8	Week 12
3943	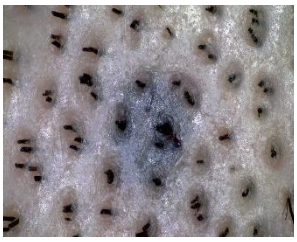	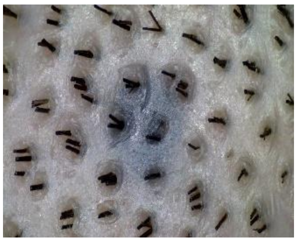	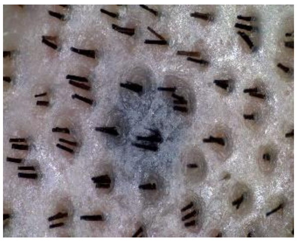	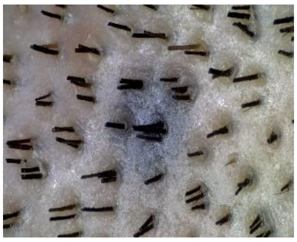
4167	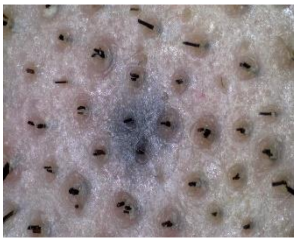	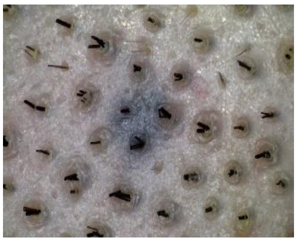	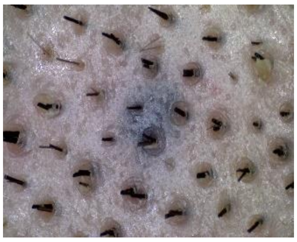	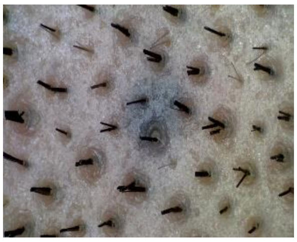
4203	-	-	-	-
3668	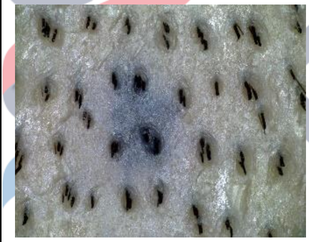	-	-	-
4005	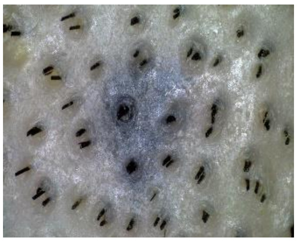	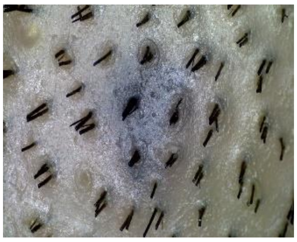	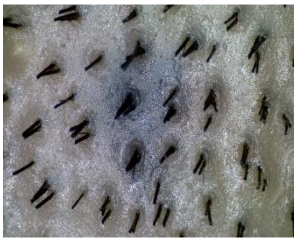	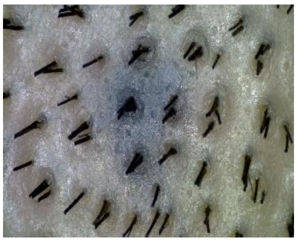
1719	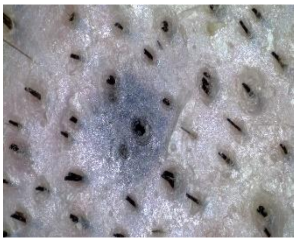	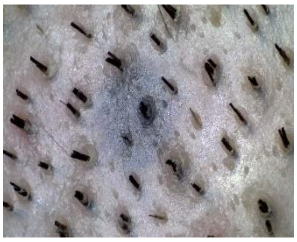	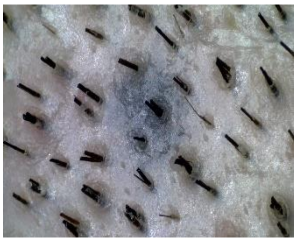	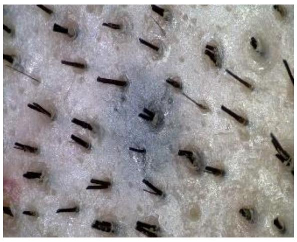

KDRI Korea Dermatology Research Institute

The contents of this report are confidential and cannot be used for other purposes unless approved.
All or any part of the contents in this report cannot be copied or published without consent of KDRI.

ID	Week 0	Week 4	Week 8	Week 12
897	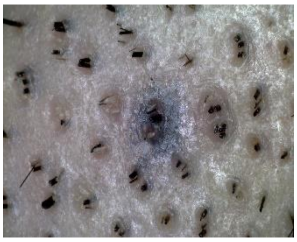	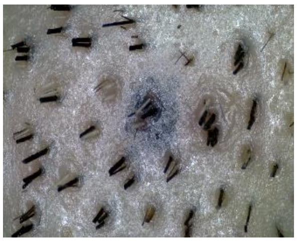	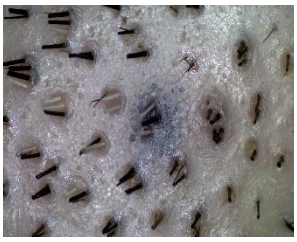	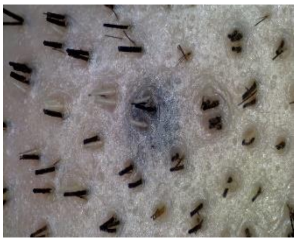
3964	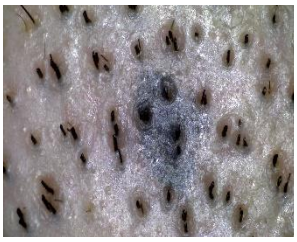	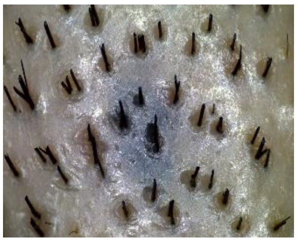	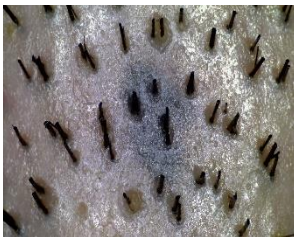	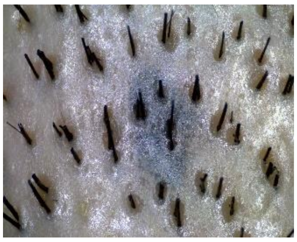
1297	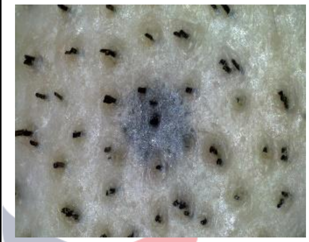	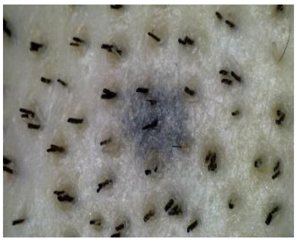	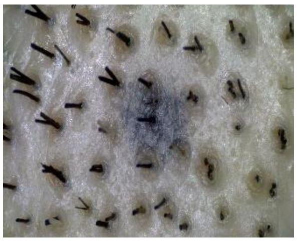	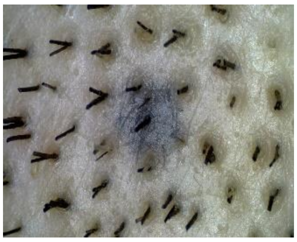
5646	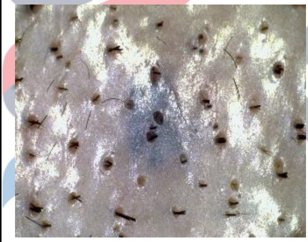	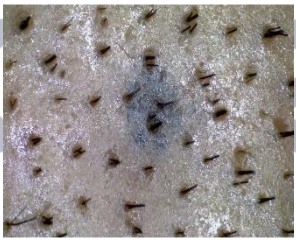	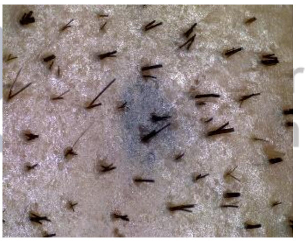	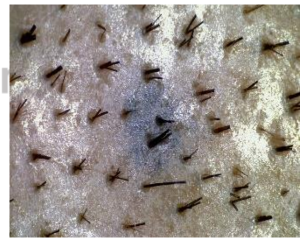
4997	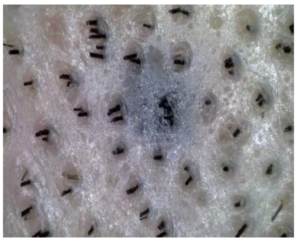	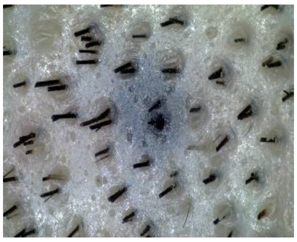	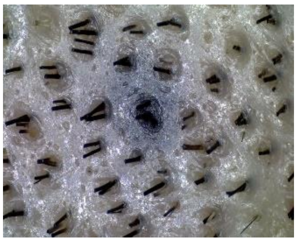	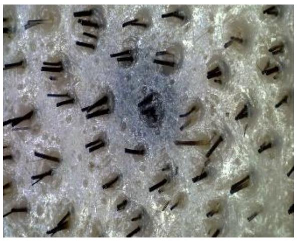

The contents of this report are confidential and cannot be used for other purposes unless approved.
All or any part of the contents in this report cannot be copied or published without consent of KDRI.

4) Phototrichogram Image –Placebo group

ID	Week 0	Week 4	Week 8	Week 12
5182	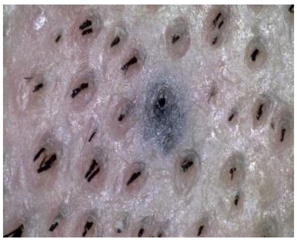	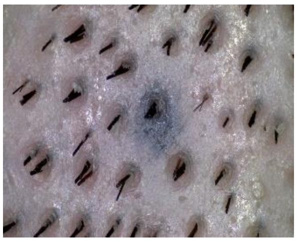	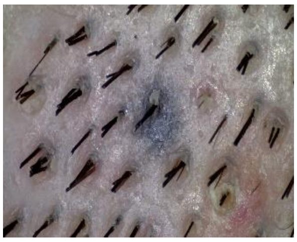	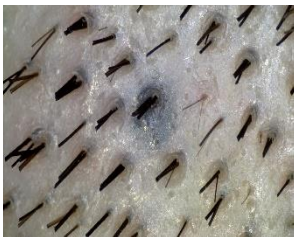
4567	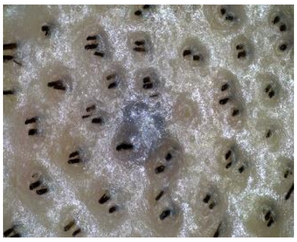	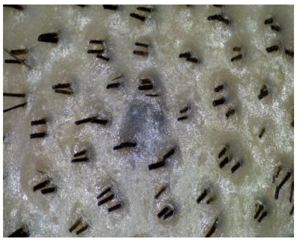	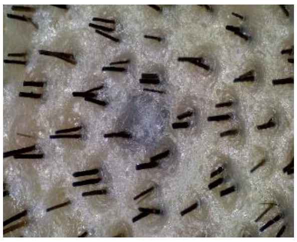	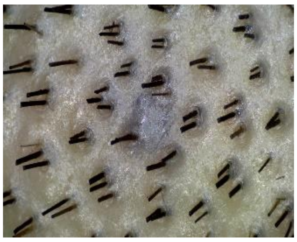
2884	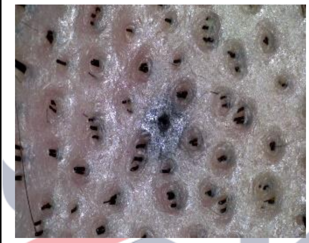	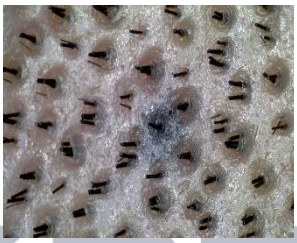	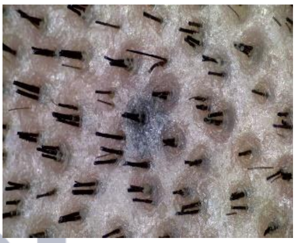	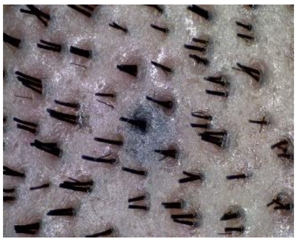
4014	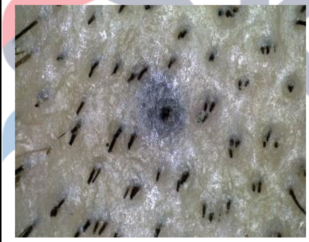	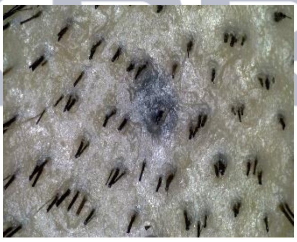	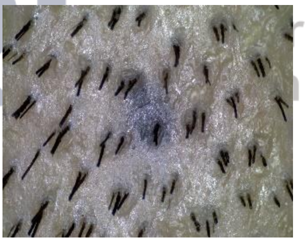	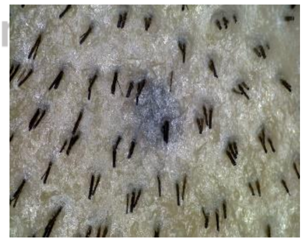
2498	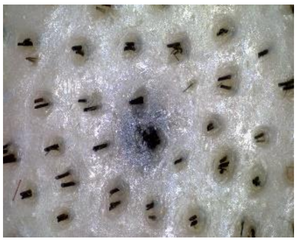	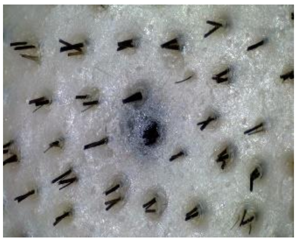	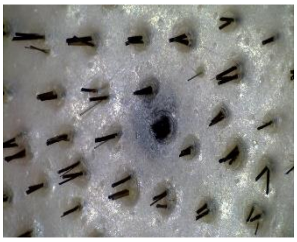	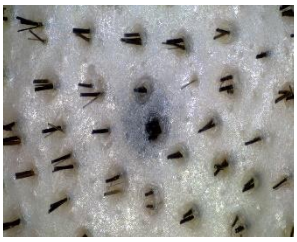
2379	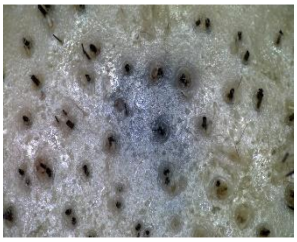	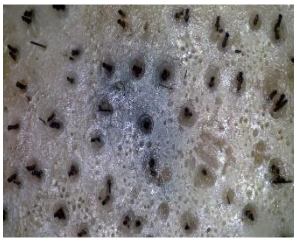	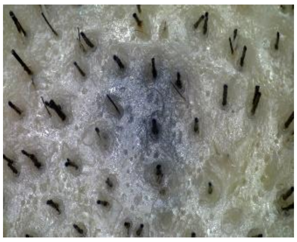	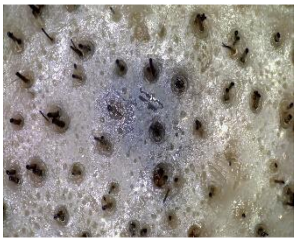

The contents of this report are confidential and cannot be used for other purposes unless approved.
All or any part of the contents in this report cannot be copied or published without consent of KDRI.

ID	Week 0	Week 4	Week 8	Week 12
763	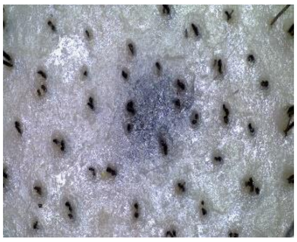	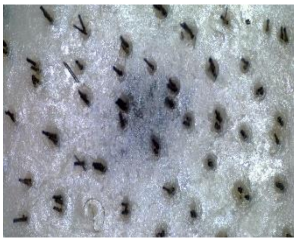	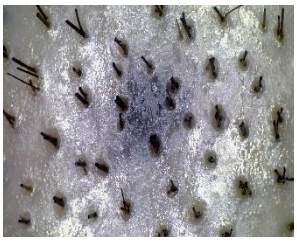	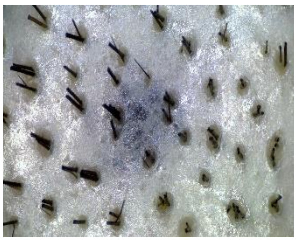
2515	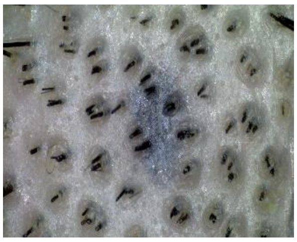	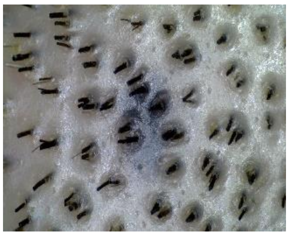	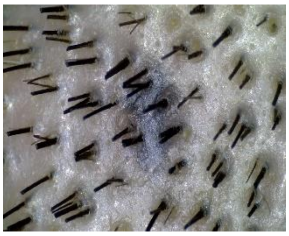	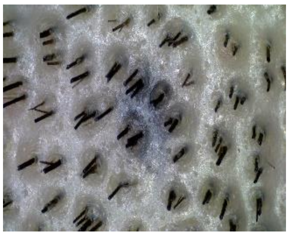
2984	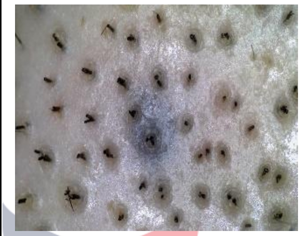	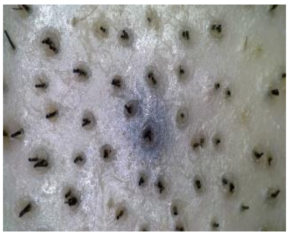	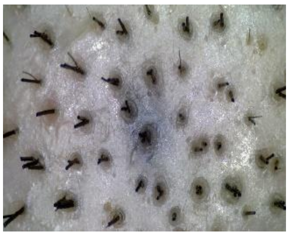	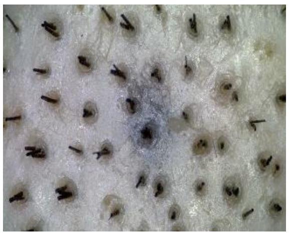
5648	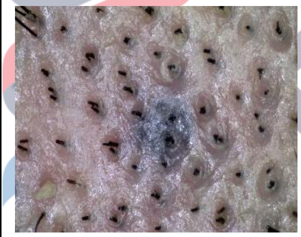	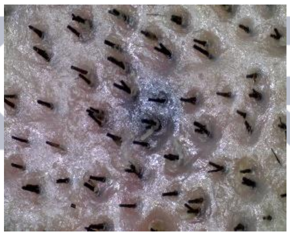	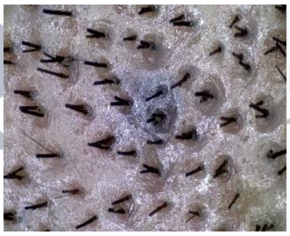	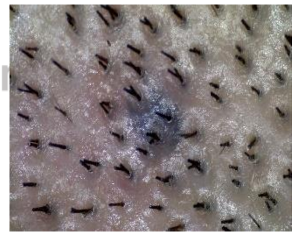
2909	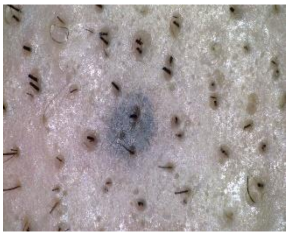	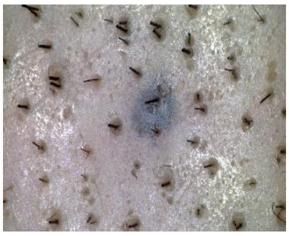	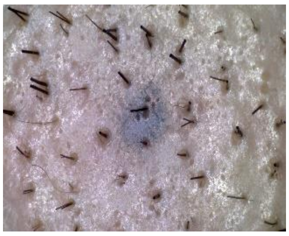	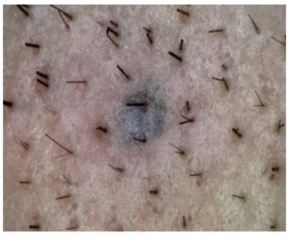
4419	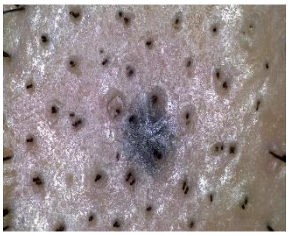	-	-	-

The contents of this report are confidential and cannot be used for other purposes unless approved.
All or any part of the contents in this report cannot be copied or published without consent of KDRI.

ID	Week 0	Week 4	Week 8	Week 12
1482	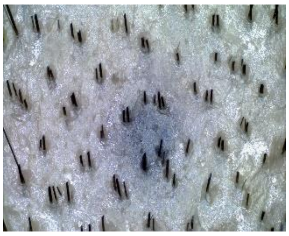	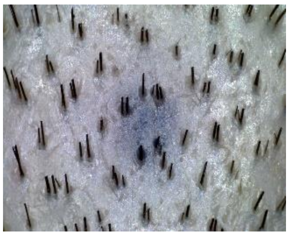	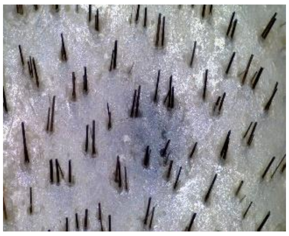	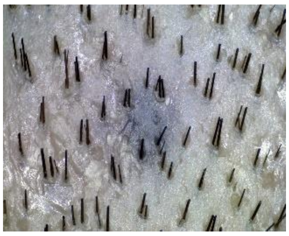
4303	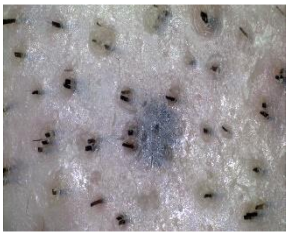	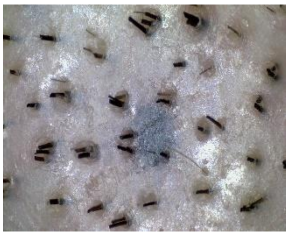	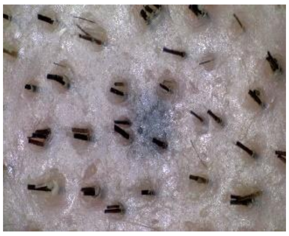	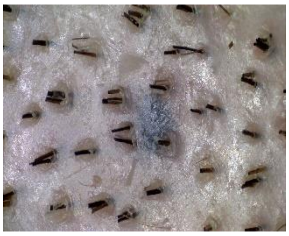
4318	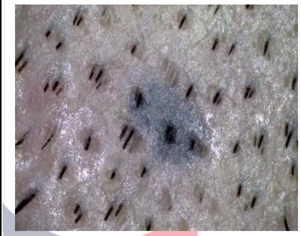	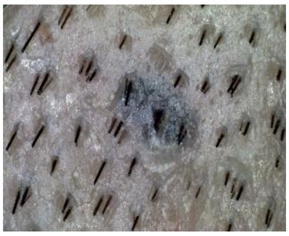	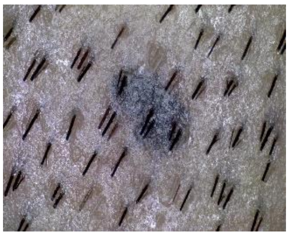	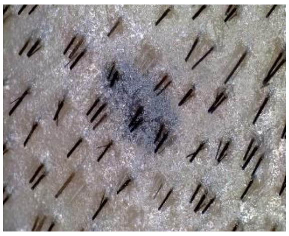
4077	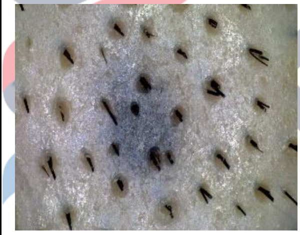	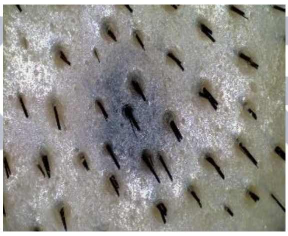	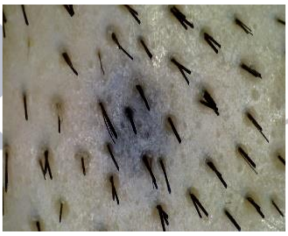	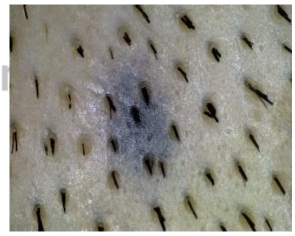
2824	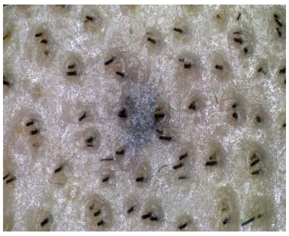	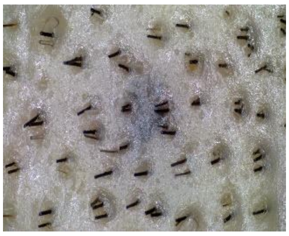	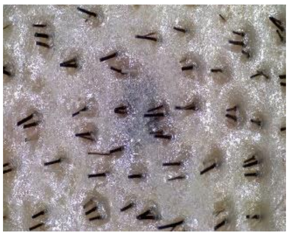	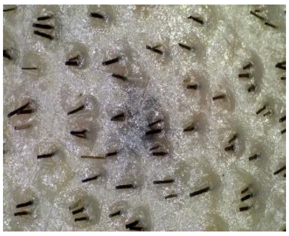

The contents of this report are confidential and cannot be used for other purposes unless approved.
All or any part of the contents in this report cannot be copied or published without consent of KDRI.
